# Tandem Cu/ZnO/ZrO_2_‑SAPO-34 System for Dimethyl Ether Synthesis from CO_2_ and H_2_: Catalyst Optimization, Techno-Economic, and Carbon-Footprint Analyses

**DOI:** 10.1021/acsengineeringau.5c00008

**Published:** 2025-04-08

**Authors:** Jasan Robey Mangalindan, Fatima Mahnaz, Jenna Vito, Navaporn Suphavilai, Manish Shetty

**Affiliations:** Artie McFerrin Department of Chemical Engineering, 14736 Texas A&M University, 100 Spence Street, College Station, Texas 77843, United States

**Keywords:** CO_2_ utilization, sustainable fuels, C−C coupling, zeolite, SAPO-34, methanol, hydrogen storage

## Abstract

To alleviate detrimental effects associated with anthropogenic emissions, the use of CO_2_ and H_2_ as feedstocks for their conversion to dimethyl ether (DME) with tandem catalysts is an attractive and sustainable route. First, we investigated the catalytic activity of bifunctional admixtures of Cu-ZnO-ZrO_2_ (CZZ) and a silicoaluminophosphate, SAPO-34, for CO_2_ hydrogenation to DME and optimized their reactivity with an emphasis on identifying optimum synthesis conditions for CZZ including Cu:Zn:Zr molar ratio and aging and calcination temperatures. The highest methanol (MeOH) productivity (10.8 mol kg_cat_^–1^ h^–1^) was observed for CZZ-611 aged at 40 °C and calcined at 500 °C. When coupled with SAPO-34, CZZ/SAPO-34 reached 20% CO_2_ conversion and 56% DME selectivity at optimized conditions (260 °C, 500 psig, and 2000 mL g_CZZ_^–1^ h^–1^) and was stable for 50 h time-on-stream, with a slight reduction in activity. Next, we performed kinetic modeling to translate lab-scale findings to industrial packed-bed reactors followed by a techno-economic analysis (TEA) with cradle-to-gate environmental footprint evaluation to evaluate its industrial applicability. A TEA of a 20,000 tpy DME plant revealed raw material costs as the main operating cost drivers (H_2_ cost comprises 47% of total cost). Considering green H_2_ ($4/kg H_2_) and captured CO_2_ as feed, the minimum DME selling price (MDSP) was $3.21/kg, ∼2.7× higher than the market price ($1.2/kg). MDSP drops to $1.99/kg with gray H_2_ ($1/kg H_2_) and fluctuates ±$0.14 with changes in CAPEX (±30%) and other economic factors. The plant’s carbon footprint was mainly affected by the H_2_ source. Green and gray H_2_ resulted in emissions of 0.21 and 4.4 kg CO_2_ eq/kg DME, respectively. Importantly, a negative carbon footprint can be achieved by using green H_2_ and CO_2_ captured directly from air. Overall, our work shows tandem catalysis as a promising approach toward sustainable DME production and identifies the pathway toward making it cost-competitive with fossil fuels.

## Graphical abstract

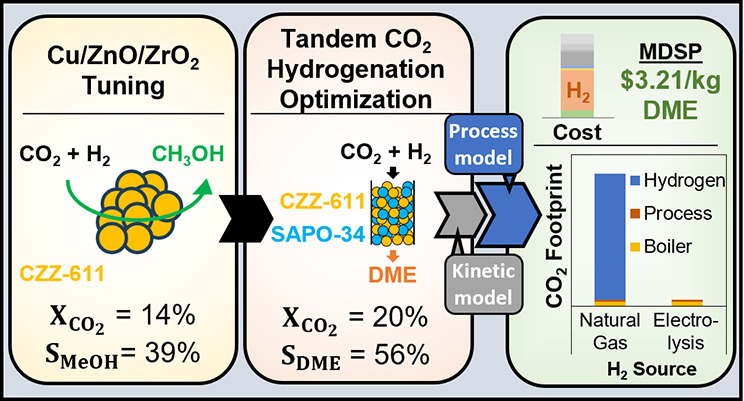

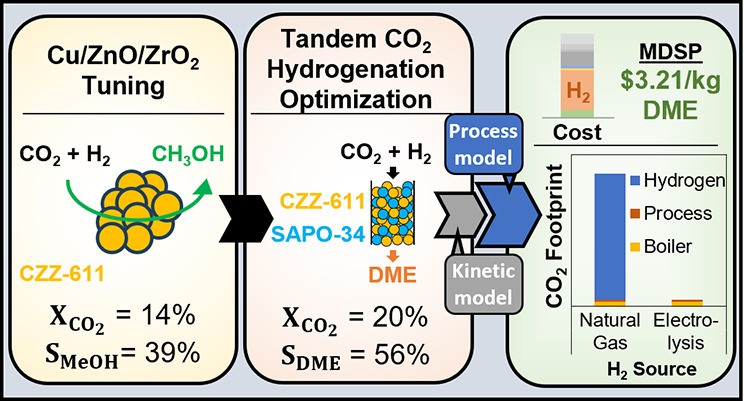

## 1 Introduction

Anthropogenic carbon dioxide (CO_2_) emissions have negatively impacted our environment, resulting in rapid climate change caused by the increase in global surface temperatures.^1^ Efforts to mitigate the effects of these emissions include the capture of CO_2_ from point sources such as industrial emissions or directly from the air, to prevent any further rise in atmospheric CO_2_ levels.^2,3^ The captured CO_2_ can be stored or used directly in chemical processes as a feedstock. Recently, interest has been drawn to the latter option, broadly classified as carbon dioxide utilization (CDU) techniques.^4,5^ Among many CDU techniques, the conversion of CO_2_ as a feedstock to produce chemicals and fuels including methane, light olefins, paraffins, and alcohols, provides a potentially cleaner production route, compared to the fossil-based sources.^6–9^ Specifically, the conversion of CO_2_ to dimethyl ether (DME) is attractive, as DME can be utilized as a clean alternative to fossil fuels and used as a hydrogen (H_2_) carrier.^4,10,11^ In 2024, DME had a market size valued at $9.5 billion and is expected to grow to $27 billion by 2037.^12^ Its nontoxic and noncarcinogenic properties together with its high cetane number, good flammability, and ability to combust with minimal soot has made it desirable as a cleaner alternative to diesel or liquified petroleum gases (LPG).^13–16^ The increasing application of DME in LPG blending or as a power plant gasoline drives the growth of the DME market.^12^ Importantly, DME can be stored and handled as a liquid, similar to LPG, as it forms a liquid phase at pressures above 0.5 MPa and, therefore, can be transported and stored using the existing infrastructure.^13^ This property makes DME more desirable as a H_2_ carrier, as it can produce H_2_ through steam reforming and potentially address the challenges associated with H_2_ transportation and storage.^4^

The catalytic conversion of CO_2_ and H_2_ to DME requires two unique active sites: (1) a metal oxide for the hydrogenation of CO_2_ to methanol (MeOH) (eq 1 below) and (2) an acid site to dehydrate MeOH to DME (eq 2).^17^ This can be achieved stepwise, via two reactors, known as the indirect method, or simultaneously in one reactor (the direct method) using physical or hybrid mixtures of the two catalysts.^18^ The direct method has been the focus of several studies as it holds specific advantages such as the integration of the reaction into one single reactor, thus reducing equipment requirements and costs.^19^ However, combining two catalysts in one reactor has its own challenges that need to be addressed.

1 $${\mathrm{CO}}_{2}\;\mathrm{hydrogenation:}\;{\mathrm{CO}}_{2}+3H_{2}⇌{\mathrm{CH}}_{3}\mathrm{OH}+H_{2}O,\Delta H=-49.5\;\mathrm{kJ/mol}$$

2 $$\mathrm{Methanol dehydration:}\;2{\mathrm{CH}}_{3}\mathrm{OH}⇌{\mathrm{CH}}_{3}O{\mathrm{CH}}_{3}+H_{2}O,\Delta H=-23.4\;\mathrm{kJ/mol}$$

3 $$\mathrm{Reverse‐water}\;\mathrm{gas shift:}\;{\mathrm{CO}}_{2}+H_{2}⇌\mathrm{CO}+H_{2}O,\Delta H=+41.2\;\mathrm{kJ/mol}$$

In a direct method, the compatibility between the metal oxide and the acidic catalyst is critical, as both operate at the same temperature (e.g., 260 °C). Hence, a metal oxide with a good activity for CO_2_ hydrogenation, for example at 260 °C, should be paired with an acidic catalyst that is active and highly selective to DME at the same temperature.^20,21^ Under the same conditions as for DME synthesis, CO_2_ can also undergo the reverse-water gas shift (rWGS) reaction (eq 3), producing carbon monoxide (CO) as a byproduct. This introduces one of the main challenges associated with DME synthesis via CO_2_, namely, controlling the yield and selectivity to DME. The endothermicity of the rWGS reaction necessitates the need for low reaction temperatures (e.g., 200–260 °C) for its suppression to achieve high DME selectivity.^4^ However, with low MeOH formation rates at these temperatures, this comes at the cost of decreased DME yields.^16^ For this reason, development of a metal oxide catalyst that shows high activity at low reaction temperatures is critical to the success of the pathway. In addition, the stability of the catalyst plays an important role. Due to the formation of water as a byproduct, the catalysts should have good resilience to water and maintain their activity for long hours of operation. Therefore, considering all these factors is important for determining and designing the tandem catalysts for the conversion of CO_2_ to DME.

One of the catalysts that is widely used for the hydrogenation of CO_2_ is Cu-ZnO-Al_2_O_3_ (CZA).^22^ Cu/ZnO-based catalysts have been shown to be effective for CO_2_ hydrogenation to MeOH especially with a structural promoter such as Al_2_O_3_ which improves reactivity.^23^ However, the hydrophilic nature of Al_2_O_3_ makes it susceptible to poisoning by water formed during the reaction.^24,25^ Recent studies have shown ZrO_2_ to be a promising support and promoter that can potentially replace Al_2_O_3_, resulting in Cu-ZnO-ZrO_2_ (CZZ), due to its weaker hydrophilic character which helps inhibit poisoning by water.^26^ The Cu/ZrO_2_ interfaces in CZZ are said to promote adsorption of CO_2_ ^24^ and improve H_2_ dissociation and atomic hydrogen (H) spillover which overall results in increased MeOH synthesis activity.^27^ Despite the better compatibility of ZrO_2_ with Cu/ZnO for the hydrogenation of CO_2_, the properties and reactivity of the catalyst still heavily rely on the synthesis methods.

Several studies have tested different synthesis procedures and conditions, which resulted in different structures and activities of the generated CZZ catalyst. The precipitation methods, aging temperature, calcination temperature, and molar composition are some of the parameters that significantly affect the final structure and activity of CZZ. For example, while Raudaskoski et al., using a coprecipitation technique, showed that longer aging times (24 h) at 80 °C after precipitation of CZZ can improve CO_2_ conversion and MeOH selectivity,^28^ Li et al. showed that calcination temperatures can affect the crystallinity and surface composition of CZZ, affecting the catalyst reactivity.^29^ Other works have also investigated the effect of the Cu/Zn/Zr ratio on the reactivity of CZZ. For example, while Huang et al. reported that CZZ synthesized through the citrate method had the most activity with a Cu:Zn:Zr mole ratio of 2.1:7.2:0.7,^30^ Witoon et al. synthesized CZZ through the reverse coprecipitation method and reported that a 4:3:3 mol ratio of Cu:Zn:Zr had the most MeOH productivity.^31^ Although reactivities similar to CZA have been achieved for CZZ, it is still unclear which set of synthesis conditions (e.g., aging temperature, calcination temperature, and CZZ composition) results in the highest yield of MeOH.

Meanwhile, the dehydration reaction is typically catalyzed by acid sites present on either alumina (Al_2_O_3_) or zeolites (e.g., HZSM-5). Particularly, zeolites have been extensively studied for CO_2_ to DME conversions as they demonstrate good stability in the presence of water (H_2_O, a product of methanol synthesis) and high activity even at moderate temperatures (200–260 °C).^32^ Most studies have focused on using medium-pore and large-pore zeolites such as FER and ZSM-5, respectively.^19,33–35^ However, given the small molecular size of DME, small pore zeolites could also be used without any detrimental effects from steric hindrance. An 8-membered ring zeotype, SAPO-34 (a silicoaluminophosphate), has shown good activity for MeOH dehydration to DME, reaching equilibrium conversions (∼88%) even at low temperatures (250 °C),^36,37^ which corroborates the potential of SAPO-34 to be applied in direct CO_2_ hydrogenation to DME. Nonetheless, to the best of our knowledge, SAPO-34 has not yet been tested for the direct (single reactor) CO_2_ to DME conversion. In addition, most studies on the direct CO_2_ to DME conversion using tandem catalysts only focus on the laboratory-scale catalytic performance.^32,34^ The economics and carbon footprint analyses of the industrial process are often disconnected from laboratory advances, with these studies often performed later, often separately by other research groups.

To that end, in this work, we report the activity of a CZZ/SAPO-34 bifunctional system for CO_2_ hydrogenation to DME and optimize the parameters and conditions that would maximize the yield of DME. This work reports the following major points: (1) synthesis parameters and composition of CZZ that maximize the CO_2_ to DME conversion, (2) effect of active site proximity between the CZZ and SAPO-34 and reaction conditions on the productivity of DME, (3) kinetic model and parameters fitting for the direct CO_2_ to DME reactions over a CZZ/SAPO-34 catalytic system, (4) economic factors that drive the CO_2_ to DME technology, and (5) a cradle-to-gate carbon footprint analysis of the process considering different feed sources. Notably, we directly investigated the kinetics of the reactions using our experimental data and applied this to a process model to generate an economic and carbon footprint analysis of the process, thereby providing a complete holistic perspective and pointing out possible challenges that this technology may face during scale-up. Taken together, this work provides a holistic picture of the CZZ/SAPO-34 system from its activity in the laboratory scale-up to its application and impacts on an industrial scale.

## 2 Materials and Methods

### 2.1 Materials

Copper nitrate hemi­(pentahydrate) (Cu­(NO_3_)_2_·2.5H_2_O, ≥99.99% trace metals basis, Sigma-Aldrich), zinc nitrate hexahydrate (Zn­(NO_3_)_2_·6H_2_O, 98%, Sigma-Aldrich), zirconium dinitrate oxide hydrate (ZrO­(NO_3_)_2_·*x*H_2_O, 99.9% trace metals basis, Bean Town Chemicals), and sodium carbonate (Na_2_CO_3_, anhydrous, >99.5% ACS, VWR Chemicals BDH) were used to synthesize CuO-ZnO-ZrO_2_ catalysts. SAPO-34 (H^+^ form) was purchased from ACS Materials in calcined form (550 °C) and was used directly. Fused α-alumina (100–200 mesh, Sigma-Aldrich) was used for spacing in the stacked catalyst bed.

### 2.2 CuO-ZnO-ZrO_2_ Synthesis

CuO-ZnO-ZrO_2_ was prepared by coprecipitation. Different compositions of CZZ were used in the study, which are denoted as CZZ-XYZ where X, Y, and Z refer to the molar ratio of Cu, Zn, and Zr, respectively. Cu­(NO_3_)_2_·2.5H_2_O, Zn­(NO_3_)_2_·6H_2_O, and ZrO­(NO_3_)_2_·*x*H_2_O corresponding to a CZZ final weight of 2.2 g were dissolved in 20 mL of deionized water (DI H_2_O). The precursor solution was mixed at 40 °C for 1 h to dissolve the metal nitrates. A 0.5 M solution of Na_2_CO_3_ in DI H_2_O was prepared separately and mixed at 40 °C until Na_2_CO_3_ was fully dissolved. A 20% excess of a Na_2_CO_3_ solution was used to ensure complete precipitation of the metals. The precursor and Na_2_CO_3_ solutions were added dropwise to 100 mL of DI H_2_O under vigorous stirring at 40 °C while maintaining a pH of 7.0–7.5 during addition. The resulting mixture was then aged for 24 h, followed by filtration under vacuum. Aging temperatures of 40 and 80 °C were tested to determine their effect on catalytic activity. The precipitates were washed excessively with DI H_2_O, dried (80 °C, 5 h), and calcined (4 h, 5 °C/min ramp rate) with air (150 mL/min) in a muffle furnace. Three temperatures were tested for calcination: 350 °C, 500 °C, and 600 °C. Finally, the calcined catalyst was homogenized using a mortar and pestle.

### 2.3 Catalyst Characterization

#### 2.3.1 Powder X-ray Diffraction (PXRD)

The PXRD patterns were obtained from a Rigaku Miniflex II X-ray instrument equipped with Cu Kα radiation (λ = 1.5406 Å). The scanning range was set from 2° to 70°, with a step rate of 0.02 and a scan rate of 1°/min.

#### 2.3.2 N_2_ Physisorption

CZZ surface areas were measured by using an Anton Paar Autosorb iQ-C-MP EPDM automated gas sorption analyzer. Nitrogen physisorption at 77 K was performed for surface area analysis by applying Brunauer–Emmett–Teller (BET) theory to the resulting adsorption–desorption isotherms. 100–150 mg of the catalyst was loaded in a 6 mm or 9 mm glass cell bulb (without a rod) and was outgassed at 350 °C for 8 h. 20 adsorption and 20 desorption points were measured ranging from *p*/*p*_0_ values of 0.05 to 0.995. BET analysis was done on adsorption data points ranging from *p*/*p*_0_ values of 0.005 to 0.3. Total pore volume (PV) was calculated based on the assumption that at relative pressures near unity the pores filled with liquid, which follows eq 4 where *P*_a_ is the pressure, *V*_ads_ is the volume of adsorbed gas, *V*_m_ is the molar volume, *T* is the temperature, and *R* is the universal gas constant.

4 $$\begin{array}{c} \mathrm{PV}=\frac{P_{a}V_{\mathrm{ads}}V_{m}}{RT} \end{array}$$

#### 2.3.3 H_2_ Temperature-Programmed Reduction (H_2_ TPR)

H_2_ TPR was performed in a quartz u-tube with ∼50 mg of sample. The sample was initially heated to 350 °C (10 °C/min ramp rate) under 50 mL/min of N_2_ for 2 h. After cooling to 40 °C, 50 mL/min of 10% H_2_/N_2_ mixture was flowed over the catalyst for 30 min and then heated to 500 °C (10 °C/min ramp rate). The outlet concentration of H_2_ was analyzed with an SRS Universal Gas Analyzer (UGA).

#### 2.3.4 CO_2_ Temperature-Programmed Desorption (CO_2_ TPD)

CO_2_ TPD was performed in a quartz u-tube with ∼90 mg of sample. The sample was initially heated to 300 °C (10 °C/min ramp rate) under a 50 mL/min 10% H_2_/N_2_ mixture for 2 h. After being cooled to 40 °C, the surface was saturated with CO_2_ at 25 mL/min. The sample was then purged with 25 mL/min of N_2_ for 1.5 h and then heated to 400 °C (10 °C/min ramp rate) under N_2_. The outlet concentration of CO_2_ was analyzed with an SRS UGA.

#### 2.3.5 Thermogravimetric Analysis (TGA)

Thermogravimetric analysis was conducted with a TA Instruments Thermogravimetric Analyzer (TGA) Q5000 IR with an infrared furnace fed with Ultra-Zero air with 100 μL platinum pans rated to 750 °C (sample weight: ∼10–20 mg, drying phase: 10 °C/min ramp to 120 °C followed by a 15 min isothermal phase, 20 mL/min air; oxidation phase: 10 °C/min ramp to 750 °C, followed by a 5 min isothermal phase).

### 2.4 Catalytic Activity Study

The hydrogenation of CO_2_ to DME was performed in a high-pressure tubular stainless-steel fixed-bed reactor. A brass heating block around the reactor tube ensured an isothermal zone where the catalysts were positioned. The catalyst bed is loaded with 0.5 g of CZZ catalyst and 0.5 g of SAPO-34, unless otherwise specified. Before each test, the catalysts were reduced *in situ* under a 100 mL/min flow of 5% H_2_ (balance N_2_) for 1 h at 300 °C and atmospheric pressure and cooled to 40 °C before the reaction. Four different bed configurations were tested as follows:

a) Dual bed: Powdered CZZ and SAPO-34 were separately pressed, crushed, and sieved into 30–60 mesh (250–595 μm). The CZZ and SAPO-34 granules were stacked as separate beds with fused α-alumina (0.5 g) in between.
b) Granule-mixed: Powdered CZZ and SAPO-34 were separately pressed, crushed, and sieved into 30–60 mesh (250–595 μm). The CZZ and SAPO-34 granules were physically mixed and stacked as a single bed.
c) Powder-mixed: Powders of CZZ and SAPO-34 were separately ground in an agate mortar and pestle for 15 min. The powders were then mixed, pressed, crushed, and sieved into 30–60 mesh (250–595 μm) to create mixed CZZ/SAPO-34 granules stacked as a single bed.
d) Mortar-mixed: Powders of CZZ and SAPO-34 were mixed and ground in an agate mortar and pestle for 15 min. The powders were pressed, crushed, and sieved into 30–60 mesh (250–595 μm) to create mixed CZZ/SAPO-34 granules stacked as a single bed.

The products were analyzed with an online gas chromatograph (Agilent 8890) equipped with a thermal conductivity detector (TCD) and a flame ionization detector (FID). An HP-PLOT Q column was connected to the detectors, where CO_2_ and CO were analyzed from the TCD while MeOH and DME were analyzed from the FID. An average of three measurements was taken for each data point with their standard deviations depicted as error bars.

The CO_2_ conversion (*X*_CO_2__) and product selectivity (*S*_*i*_) were computed on a molar carbon (mol_C_) basis and were calculated through the following equations:

5 $$\begin{array}{c} {\mathrm{CO}}_{2}\;\mathrm{Conversion},\;X_{{\mathrm{CO}}_{2}}=\frac{\sum v_{i}C_{i}}{C_{{\mathrm{CO}}_{2,\mathrm{out}}}+\sum v_{i}C_{i}} \end{array}$$

6 $$\begin{array}{c} \mathrm{Selectivity},\;S_{i}=\frac{v_{i}C_{i}}{\sum {v_{i}C}_{i}} \end{array}$$

where C_CO_2,out__ is the concentration of CO_2_ at the outlet, *v*_*i*_ is the number of carbons in product *i*, and *C*_*i*_ is the concentration of product *i* at the outlet.

Gas hourly space velocity (GHSV, mol g_CZZ_^–1^ h^–1^) was defined based on the mass of the CZZ catalyst as shown in eq 7. This was calculated relative to CZZ as the CO_2_ conversion was observed to be mostly dependent on the amount of CZZ catalyst in the experiments conducted. This helped obtain experimental data with similar CO_2_ conversion, even with varying weights of SAPO-34.

7 $$\begin{array}{c} \mathrm{GHSV}=\frac{Q_{\mathrm{inlet}}}{w_{\mathrm{CZZ}}}\times 60 \end{array}$$

where *Q*_inlet_ is the inlet volumetric gas flow rate (mL min^–1^) and *w*_CZZ_ is the weight of the CZZ catalyst (g).

The space–time yield (STY, mol kg_cat_^–1^ h^–1^) of product *i* was computed using eq 8 where *ṅ*_CO2_ refers to the molar flow rate of CO_2_ in mol h^–1^, and *w*_cat_ is the weight of the catalyst in kilograms (kg_cat_).

8 $$\begin{array}{c} \mathrm{Space}\text{−}\mathrm{time Yield},\;\mathrm{STY}=\frac{{ṅ}_{{\mathrm{CO}}_{2}}X_{{\mathrm{CO}}_{2}}S_{i}}{w_{\mathrm{cat}}v_{i}} \end{array}$$

We also defined DME yield (% mol_C_/mol_C_) as the product of CO_2_ conversion (*X*_CO_2__) and DME selectivity (*S*_DME_) as shown in eq 9, which was used in comparing the results of the parametric studies.

9 $$\begin{array}{c} \mathrm{DME Yield}=X_{{\mathrm{CO}}_{2}}S_{\mathrm{DME}} \end{array}$$

### 2.5 Techno-economic and Carbon Footprint Analyses

Process design and simulation were carried out in Aspen Plus version 14 to obtain mass and energy balances. A kinetic model for the CZZ/SAPO-34 system was fitted based on Wild et al.^33^ and applied in the process model. The capital expenses (CAPEX) and operating expenses (OPEX) were then estimated. Sensitivity analysis was performed to identify the effect of key economic parameters on the minimum DME selling price (MDSP). Additionally, an evaluation of the carbon footprint based on varying sources/production methods of feedstock and utility was performed.

System boundaries and plant specifications:

- The process design focused on the production and purification of DME from CO_2_ and H_2_. CO_2_ capture and H_2_ production, and their subsequent purification and transportation, are not considered in the simulation. However, the cost and emissions from different CO_2_ and H_2_ sources were considered in the economic analysis. As a base case, it was assumed that CO_2_ and H_2_ were supplied to the plant at 30 bar and ambient temperature.
- The production of DME consisted of 1) compression, mixing, and heating of CO_2_ and H_2_ to achieve the operating temperature and pressure, 2) reaction with a catalyst in a multitube fixed bed reactor to produce DME, 3) purification of DME through condensation and distillation, and 4) recycling of unreacted gases and recovery of byproduct (MeOH).
- Utilities include HP-steam (255 °C), cooling water, and ethane refrigerant. Electricity was assumed to be supplied from the grid.
- The plant was assumed to have a capacity of 20,000 tons per year (tpy). Current DME production plants have capacities ranging from 3,000 to 10 million tpy; hence the assumed capacity falls within the range of typical industrial plants. In addition, we considered a plant lifetime of 20 years with 8000 h/year production time. Fuel-grade DME is produced as defined by ISO-16861:2015. The plant was assumed to be built in the United States.

Basis and assumptions for process design:

- Peng–Robinson equation of state was used to estimate the thermodynamic properties.^38^ This was applied to all equipment and streams.
- The compressors were assumed to operate with an isentropic efficiency of 0.85 and mechanical efficiency of 0.95.^39^
- A multitube fixed bed reactor was applied utilizing the fitted kinetic model as described above. The reactor was based on a 1D pseudohomogeneous plug flow reactor model assuming an isothermal operation with no internal and external mass transfer limitations. The reactor operates with a 3:1 ratio of CO_2_ and H_2_ at an average temperature of 260 °C and pressure of 35 bar. The catalyst weight inside the reactor was adjusted to achieve a GHSV of 2000 mL g_CZZ_^–1^ h^–1^ as defined in eq 7. Pressure drops within the reactor bed were estimated using the Ergun equation. Boiling water was used to absorb heat emitted by the reaction. The flow rate of the water was adjusted to achieve an average temperature of 260 °C across the length of the reactor.
- Heat exchangers were modeled using HeatX block applying a shortcut method on a design basis simulation. A minimum temperature difference of 10 °C was applied for heat transfer. The utilities applied were high-pressure steam (HP steam), cooling water, and ethane refrigerant depending on the heating/cooling requirements of the streams.
- The operating pressure and temperature of the flash drum were based on a sensitivity analysis to recover >90% of DME in the liquid phase.
- Distillation columns use RadFrac models in Aspen Plus. The process was initially modeled using DSTWU units to estimate the number of trays, reflux ratio, feed position, and distillate-to-feed ratio. The distillation columns were later changed to more rigorous RadFrac models and further optimized to achieve the desired purity of products.

Basis and assumptions for economic analysis:

- Purchased equipment cost (PEC) was obtained from Aspen Process Economic Analyzer (APEA), and the capital expenditures (CAPEX) were estimated using Lang factors as shown in Table S1.
- Operational expenditures (OPEX) were divided into variable, fixed, plant overhead, and general costs. Variable expenses include feedstock (CO_2_ and H_2_), utilities, annualized cost of catalyst (catalyst lifetime of 2 years was assumed), labor and supervision, maintenance, operating supplies, laboratory charges, and royalties. Fixed expenses include taxes and insurance. General expenses include administrative costs, distribution and marketing, and research and development. The costs assumed for feedstock and utilities are listed in Table 1. The methodology for calculating OPEX is shown in Table S2.^40^

**Table 1:** Assumed Economic Parameters Including Prices of Feedstock and Utilities

	Value	Unit	Reference
H_2_	4	$/kg	^41^
CO_2_	0.1	$/kg	^42^
CZZ	23	$/kg	SI section 7.2.1
SAPO-34	188	$/kg	SI section 7.2.2
Natural gas	4.59	$/1000 ft^3^	^43^
Electricity	0.166	$/kWh	^44^
Cooling water	0.06	$/m^3^	^45^
Methanol	0.66	$/kg	^46^

The assumed financial parameters are given in Table 2. The plant was assumed to be in the United States with 2022 as the base year of the study. The plant was financed through a combination of debt (*D*) and equity (*E*) with a ratio of 1:1, considering a debt interest rate (*i*_D_) of 6% and a cost of equity (*i*_E_) of 9%. A straight-line depreciation method was applied with assets depreciating within 10 years. We considered a 3-year construction period, spending 20%, 50%, and 30% on the first, second, and third years, respectively. A 50% operating capacity was realized for the first year of operation, 90% for the second year, and 100% for the succeeding years. The plant was assumed to have an operating life of 20 years and is fully depreciated at its end of life (no salvage value).

**Table 2:** Economic Parameters Are Assumed

	Value	Unit
Location	United States	-
Base year	2022	-
Construction period	3	year
Plant operating life	20	year
Plant operating hours	8000	h/year
Tax rate	25	%
Debt-to-equity ratio (*D*/*E*)	1:1	-
Debt interest rate (*i*_*D*_)	6	%
Cost of equity (*i*_E_)	9	%
WACC	7.5	%
Depreciation method	Straight line	-
Depreciation period	10	y
Salvage value	0	$

The annual DME production costs (ADPC) were calculated as the sum of the annualized CAPEX (CAPEX_Annual_) and OPEX. The calculation for CAPEX_Annual_ is shown in eq 10.

10 $$\begin{array}{c} {\mathrm{CAPEX}}_{\mathrm{Annual}}=\mathrm{CAPEX}\left(\frac{r_{d}\times {\left(1+r_{d}\right)}^{n}}{{\left(1+r_{d}\right)}^{n}-1}\right) \end{array}$$

where *r*_d_ is the discount rate and *n* is the plant operating life. The weighted average cost of capital (WACC) was considered as the discount rate, which was calculated using eq 11.

11 $$\begin{array}{c} \mathrm{WACC}=\left(\frac{D}{D+E}\right)i_{D}+\left(\frac{E}{D+E}\right)i_{E} \end{array}$$

MDSP was estimated for a net present value (NPV) = 0 or equivalently when the internal rate of return (IRR) = *r*_d_. The discounted cash flow model is represented in eqs 12 and 13.

12 $$\begin{array}{c} \overset{20}{\underset{n=1}{\sum }}\frac{{\mathrm{CF}}_{n}}{{\left(1+\mathrm{IRR}\right)}^{n}}=0 \end{array}$$

13 $$\begin{array}{c} {\mathrm{CF}}_{n}=P_{n}\left(1-t\right)+d_{n}t \end{array}$$

where CF_*n*_ is the cash flow; *P*_*n*_ is the profit; and *d*_*n*_ is the depreciation at year *n*.

#### 2.5.1 Carbon Footprint Analysis

The carbon footprint of the DME plant was evaluated in terms of the net CO_2_ emissions (CO_2,net_, kg of CO_2_ eq/kg of DME) as described in eq 14. CO_2,raw materials_ (kg CO_2_ eq/kg DME) refers to emissions associated with the production/capture of raw materials. Process CO_2_ emissions (CO_2,process_, kg CO_2_ eq/kg DME) include unreacted CO_2_ and product gases in the purge stream. Lastly, CO_2,utilities_ (kg CO_2_ eq/kg DME) are emissions from the combustion of natural gas in generating high pressure steam. Details of the assumed emission intensity of each component are listed in Table S3.

14 $$\begin{array}{c} {\mathrm{CO}}_{2,\mathrm{net}}={\mathrm{CO}}_{2,\mathrm{raw materials}}+{\mathrm{CO}}_{2,\mathrm{process}}+{\mathrm{CO}}_{2,\mathrm{utilities}} \end{array}$$

## 3 Results

### 3.1 Catalytic Performance of CZZ for MeOH Synthesis

The synthesis parameters of CZZ can greatly affect its textural and physicochemical properties which may ultimately affect its ability in converting CO_2_ to MeOH. The synthesis method applied in this work utilizes a coprecipitation technique.^28^ To optimize the activity of CZZ, synthesis using different aging (40 and 80 °C) and calcination temperatures (350 °C, 500 °C, and 600 °C) were investigated as these parameters can significantly impact the crystallinity and surface structure of the precipitated catalyst.^27,28^ Based on detailed catalyst testing, an aging temperature of 40 °C and calcination temperature of 500 °C resulted in the CZZ with the highest MeOH yield. Details of this can be found in Section S2 of the Supporting Information (SI). The optimum aging and calcination temperatures were then used in synthesizing CZZ catalysts with varying compositions, which played a major role in the conversion of CO_2_ to MeOH. We note that all catalyst evaluations were carried out with negligible mass-transfer limitations (see Section S4 in SI).

The different compositions of CZZ and their corresponding MeOH yields were investigated and are shown in Table 3. The catalysts are denoted as CZZ-*XYZ* where *X*, *Y*, and *Z* refer to the molar ratios of Cu, Zn, and Zr, respectively. The Cu:Zn ratio was initially varied among 2:5, 4:3, and 6:1 while maintaining the Zr molar ratio at 3 (CZZ-253, CZZ-433, and CZZ-613, respectively). The Zr content was then adjusted (CZZ-611, CZZ-613, and CZZ-615) with a constant Cu:Zn ratio of 6:1. These ranges of compositions tested were based on compositions used in previous studies.^29–31,34,47^ The reactivity of the five unique compositions of CZZ (summarized in Table S5) was investigated at 240 °C, 500 psig, and 18000 mL g_CZZ_^–1^ h^–1^, and the results are summarized in Table 3. Data for reaction temperatures of 220 °C, 260 °C, and 280 °C are also available in Figure S4. Across all temperatures, it was observed that MeOH yield goes up with increasing Cu content of CZZ which suggests that Cu plays an important role in MeOH synthesis. To further identify how Cu participates in the reaction, CuO/SiO_2_ (at room temperature) and ZnZrO_*x*_ supports were tested at the same conditions. Both CuO/SiO_2_ and ZnZrO_*x*_ showed minimal activity for MeOH synthesis implying that Cu on its own is not efficient in producing MeOH, and MeOH formation most likely happens in the Cu/Zn and/or Cu/Zr interface, in line with previous works.^24,27,48,49^

**Table 3:** Properties and Catalytic Activity (240 °C, 500 psig, 18000 mL g_CZZ_^–1^ h^–1^) of CZZ with Different Compositions

Catalyst	*X*_CO_2__^a^^,^^i^ (% mol_C_/mol_C_)	*S*_MeOH_^b^^,^^i^ (% mol_C_/mol_C_)	MeOH STY^c^ ^,^ ^i^ (mol kg_cat_^–1^ h^–1^)	*d*_CuO_^d^ (nm)	*S*_BET_ (m^2^/g)	H_2_ consumed^e^ (mol H_2_/kg_cat_)	CO_2_ desorbed^f^ (μmol CO_2_/g_cat_) [μmol CO_2_/g_Cu_]
CZZ-253	7.9	44.0	6.9	12.8	84.9	4.0	99.0 [729]
CZZ-433	10.0	37.9	7.6	11.5	73.8	6.6	93.8 [344]
CZZ-611	13.7	39.2	10.8	8.1	59.5	12.8	74.8 [134]
CZZ-613	12.0	37.2	8.9	9.1	71.8	10.5	83.1 [202]
CZZ-615	10.3	41.5	8.6	9.4	82.0	8.5	75.0 [231]
CuO/SiO_2_	1.5	37.8	1.1	16.6	143.6	9.3	n.d.^h^
ZnZrO_*x*_	0.1	–^g^	0.2	-	48.1	0.9	46.7

a CO_2_ conversion – calculated as the total moles of carbon (mol_C_) in products divided by the total mol_C_ in the outlet stream (unreacted CO_2_ + products).

b Methanol selectivity – calculated as mol_C_ in MeOH divided by the total mol_C_ of products.

c Methanol space–time yield – calculated by dividing the production rate of MeOH (mol h^–1^) by the weight of the catalyst (kg_cat_).

d Calculated using the Scherrer equation for the (111) facet of CuO.

e Calculated from H_2_ TPR.

f Calculated from CO_2_ TPD.

g Only MeOH was detected by the gas chromatograph, but it is possible that a small amount of CO exists which is below the detection limit of the thermal conductivity detector.

h Not detected.

i Carbon balances were within ± 3%.

To explain the reactivity differences, we looked into the powder X-ray diffraction (PXRD) patterns, textural properties, catalyst reducibility, and CO_2_ adsorption ability of the calcined catalysts. Figure 1 shows the PXRD patterns of the CZZ catalysts of different compositions. Peaks for CuO were apparent in all samples, but as Cu loading decreased, these peaks became less well-defined. Interestingly, ZnO peaks were only visible in samples with the highest Zn loadings (CZZ-253 and CZZ-433), and ZrO_2_ peaks were not visible in any CZZ samples, indicating a high dispersion or amorphous phase of Zr. Given that the activity was mostly attributed to Cu, we estimated the crystallite sizes of CuO particles using the Scherrer equation (for the peak of the (111) plane of CuO (*2ϑ* = 38.8°)). Looking at the crystallite sizes, higher MeOH yields were achieved with smaller CuO crystallite sizes. This is likely due to the increased number of Cu/Zn and Cu/Zr interfaces due to smaller Cu crystallite sizes. The peak associated with CuO broadened with increasing Zr incorporation (CZZ-611 to 615) indicating smaller crystallite sizes for CuO. Furthermore, lower surface areas of CZZ were observed with higher Cu content. However, the activity did not appear to vary directly with crystallite size and surface area.

**Figure 1:**
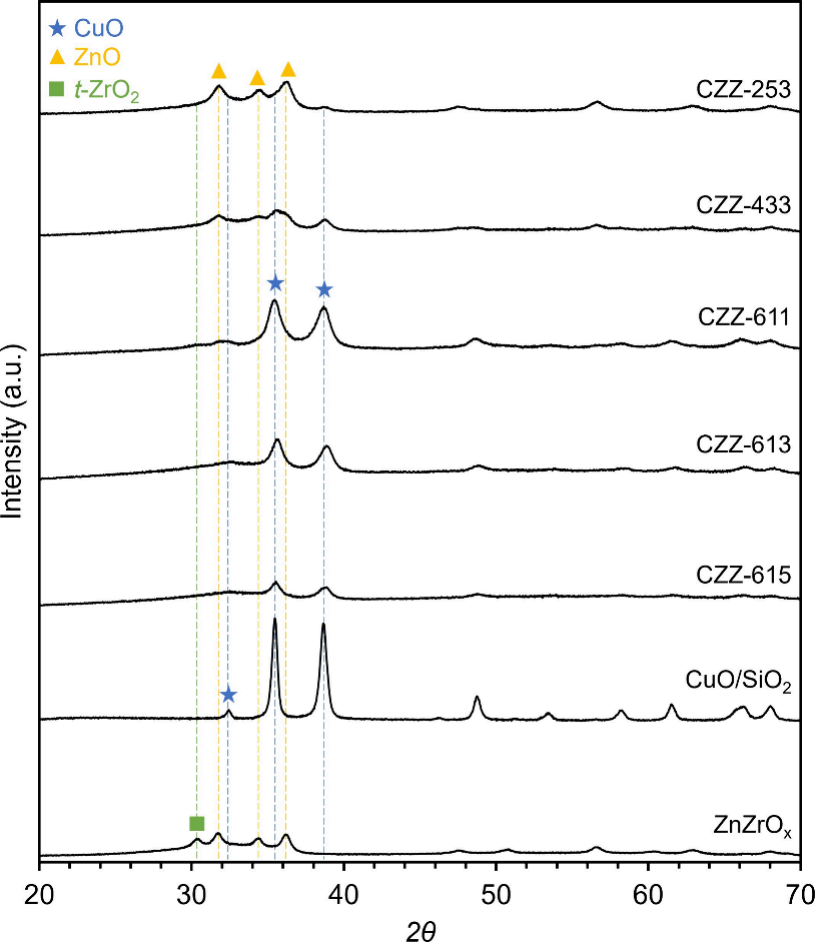
Powder X-ray diffraction (PXRD) patterns of calcined CZZ with different Cu:Zn:Zr molar ratios together with the PXRD pattern of CuO/SiO_2_ and ZnZrO_*x*_. Blue stars, yellow triangles, and green squares represent CuO, ZnO, and t-ZrO_2_ diffraction peaks, respectively. Catalysts and their compositions (Cu:Zn:Zr:Si % mol/mol) from top to bottom: CZZ-253 (20:50:30:0), CZZ-433 (40:30:30:0), CZZ-611 (75:12.5:12.5:0), CZZ-613 (60:10:30:0), CZZ-615 (50:8.3:41.7:0), CuO/SiO_2_ (48.6:0:0:51.4), and ZnZrO_*x*_ (0:50:50:0). CZZ and ZnZrO_*x*_ catalysts were synthesized via coprecipitation with an aging temperature of 40 °C and calcination temperature of 500 °C. CuO/SiO_2_ was synthesized via the wet impregnation method and followed the same drying and calcination procedures as CZZ.

To characterize the reducibility of the catalysts, H_2_ temperature-programmed reduction (H_2_ TPR) was performed on the calcined catalysts as shown in Figure S5, and the corresponding H_2_ consumptions are summarized in Table 3. A linear correlation between the Cu content of the catalyst and H_2_ consumption was observed (Figure 2A) with an H_2_/Cu molar ratio of ∼1.37, which is close to the theoretical value of 1.0 for the reduction of CuO to Cu metal. There is minimal reduction observed for ZnZrO_*x*_, implying that H_2_ consumption is most likely associated with the reduction of CuO in the CZZ catalyst. The slightly higher H_2_/Cu molar ratio could be attributed to the reduction of mixed metallic oxides (e.g., Cu–Zr–O_*x*_) formed at the Cu/Zn and Cu/Zr interfaces, in addition to the partial reduction of ZnZrO_*x*_. Taken together, the MeOH yield of CZZ was found to be directly correlated with the reducibility of the catalyst (Figure 2B). Catalysts with greater reducibility could indicate the existence of more oxygen vacancies which serve as actives sites for the reaction.^48^ The slope of Figure 2B was 0.42, which could serve as an indication of the activity of the active site, corresponding to 0.42 mol of MeOH produced per hour for every mole of active site. However, this does not explain why CuO/SiO_2_, which had high reducibility, did not give high MeOH yields. Thus, we performed CO_2_ temperature-programmed desorption (CO_2_ TPD) (Figure S6) and found that Cu supported in SiO_2_ did not show significant CO_2_ desorption (refer to Table 3) which explains its low activity for CO_2_ hydrogenation. CZZ catalysts on the other hand showed significant CO_2_ adsorption behavior, ranging from 75 to 100 μmol CO_2_/g_cat_, which demonstrated that the Cu/metal oxide interfaces likely promote the adsorption of CO_2_ (*vide infra*), resulting in higher activities. However, the CO_2_ adsorption ability of the catalysts was not directly related to Cu content (refer to Table 3 CO_2_ desorption values in square brackets) which further proves that some “synergy” among Cu, Zn, and Zr caused the improvement in MeOH synthesis activity. Examples of the “synergy” include the formation of Cu/Zn or Cu/Zr surface alloys, tuning of H_2_ dissociation, change in the adsorption of CO_2_, and modification of surface properties such as basicity or defect concentrations which ultimately improve activity to MeOH production.^49^ Overall, CZZ-611 had the highest MeOH yield, which was used for the tandem catalyst studies.

**Figure 2:**
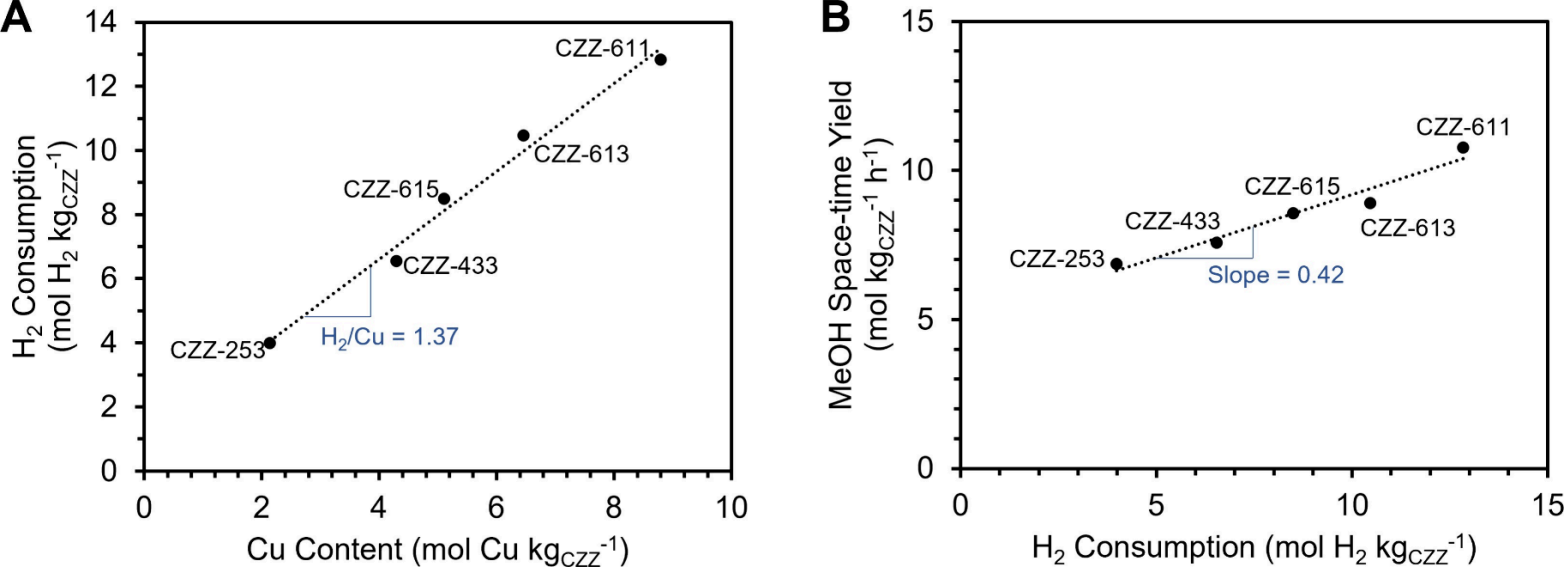
A) H_2_ consumption during H_2_ TPR (mol H_2_ kg_CZZ_^–1^) vs Cu content of CZZ catalyst (mol Cu kg_CZZ_^–1^). B) Methanol space–time yield (MeOH STY) (mol kg_CZZ_^–1^ h^–1^) vs H_2_ consumption during H_2_ TPR (mol H_2_ kg_CZZ_^–1^). The catalysts are denoted as CZZ-*XYZ* where *X*:*Y*:*Z* refers to the Cu:Zn:Zr molar ratio.

### 3.2 Catalytic Performance of the CZZ/SAPO-34 Tandem System

#### 3.2.1 Effect of Bed Configuration

The catalytic performance of the CZZ/SAPO-34 tandem system was initially assessed at a temperature of 260 °C, pressure of 500 psig, and GHSV of 18000 mL g_CZZ_^–1^ h^–1^. We investigated the performance of a CZZ single bed (SB) and four different bed configurations of the tandem system: dual bed (DB), granule-mixed (GM), powder-mixed (PM), and mortar-mixed (MM) (Figure 3) which compared how the proximity of the metal and acidic active sites affected the formation of DME.

**Figure 3:**
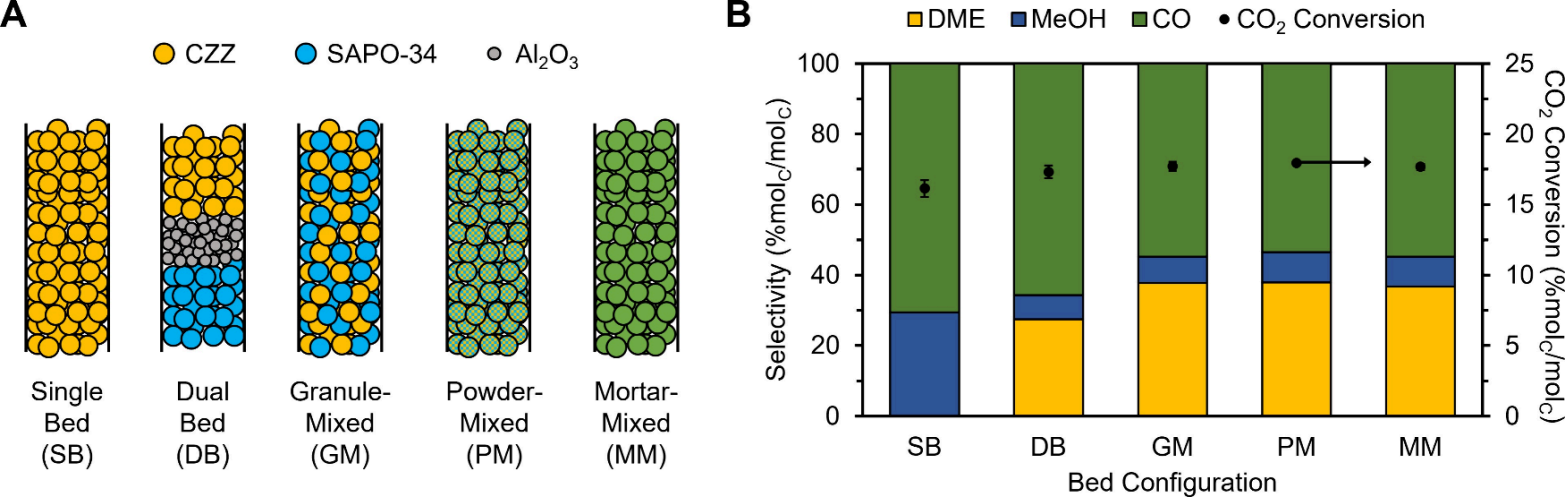
A) Schematic of bed configurations used in the study. Yellow, blue, and gray spheres refer to CZZ, SAPO-34, and Al_2_O_3_, respectively. Green spheres refer to the mixture of CZZ and SAPO-34. From left to right: single bed (SB) consisting of CZZ only, dual bed (DB) with CZZ as top bed and SAPO-34 as bottom bed, granule-mixed (GM) with pellet mixture of CZZ and SAPO-34, powder mixed (PM) with pellets formed from lightly mixed powders of CZZ and SAPO-34, and mortar mixed (MM) with pellets formed from CZZ and SAPO-34 mixed using a mortar and pestle. Feed gases flow from the top to bottom. B) Catalytic performance of the CZZ/SAPO-34 tandem system of the different bed configurations. The left axis shows product selectivity (% mol_C_/mol_C_) with yellow, blue, and green bars representing DME, MeOH, and CO, respectively. The right axis shows the CO_2_ conversion (% mol_C_/mol_C_) represented by black circles. Reaction conditions: 260 °C, 500 psig, 18000 mL g_CZZ_^–1^ h^–1^, H_2_:CO_2_ ratio = 3:1, mass of CZZ = 0.5 g, CZZ:SAPO-34 mass ratio = 1:1. Carbon balances were within ±3% error.

First, we compared the activities of CZZ only (SB) and the DB tandem system. With only CZZ, CO_2_ was converted into CO and MeOH via the reverse water–gas shift (rWGS) and CO_2_ hydrogenation, respectively.^50^ At 260 °C, a CO_2_ conversion of ∼16% with a MeOH selectivity of ∼29% and a CO selectivity of ∼71% was achieved. Coupling the CZZ with SAPO-34 in a DB configuration resulted in the conversion of MeOH to DME on the acidic sites of SAPO-34 through a dehydration mechanism.^50^ A synergetic effect can be observed when the two catalysts are placed together in the bed at 260 °C with a slight increase in CO_2_ conversion (∼17%) and a decrease in CO selectivity (∼66%). The presence of SAPO-34 in the bed increased the overall equivalent MeOH selectivity (MeOH + DME) from ∼29% in the SB to ∼34% in the DB. Looking at the distribution of MeOH and DME in the DB, ∼80% of MeOH produced from CZZ was converted into DME suggesting that SAPO-34 functions as an effective dehydration catalyst. In addition, no other byproducts (hydrocarbons or other oxygenates) were observed showing that SAPO-34 is a highly selective dehydration catalyst for DME production as previously reported.^37^ With this, the question now arises whether increasing the proximity between the active sites on CZZ and the acidic sites would further improve the yield of DME.

We investigated the activity at bed configurations that have closer proximity of active sites by mixing CZZ and SAPO-34 as pellets (GM) or powders (PM and MM). The PM and MM configurations have roughly the same active site distances but are only varied in the preparation method. PM uses a lightly mixed catalyst mixture unlike the mortar-mixed catalyst mixture (MM) which grinded the catalysts, allowing solid-state ion exchange of metal ions with the Brønsted acid sites of SAPO-34.^51^ At 260 °C, GM, PM, and MM configurations had DME + MeOH selectivity of ∼46%, higher than the DME + MeOH selectivity from the DB configuration (∼34%) at the same CO_2_ conversions (∼18%). This suggests that the close contact of the active sites between CZZ and SAPO-34 in GM, PM, and MM improved the yield to DME and MeOH. This could be due to the faster transfer of intermediates between the active sites of CZZ and SAPO-34, allowing faster conversions of methanol and shifting the equilibrium toward further MeOH and DME production.^52^ When comparing the results among GM, PM, and MM, the closer proximity of active sites did not show changes in activity, suggesting that the transfer of intermediates (between active sites and inside the catalyst) in GM does not limit the reaction. It is to be noted that previous works have reported reduced activity at very close proximity (MM) owing to the ion exchange of metal cations with the acidic sites of the zeolite which were not observed in our results, likely due to the lower reaction temperature employed in this study.^52,53^ Due to the enhanced selectivity to DME with GM configuration and no further improvements with PM and MM, GM was chosen for further parametric studies.

#### 3.2.2 Parametric Studies

In order to determine conditions that maximize the DME yield of the CZZ/SAPO-34 tandem system, the temperature, pressure, GHSV, H_2_:CO_2_ ratio, and CZZ:SAPO-34 mass ratio were varied. Figure 4 shows the activity of the tandem system at different varied parameters.

**Figure 4:**
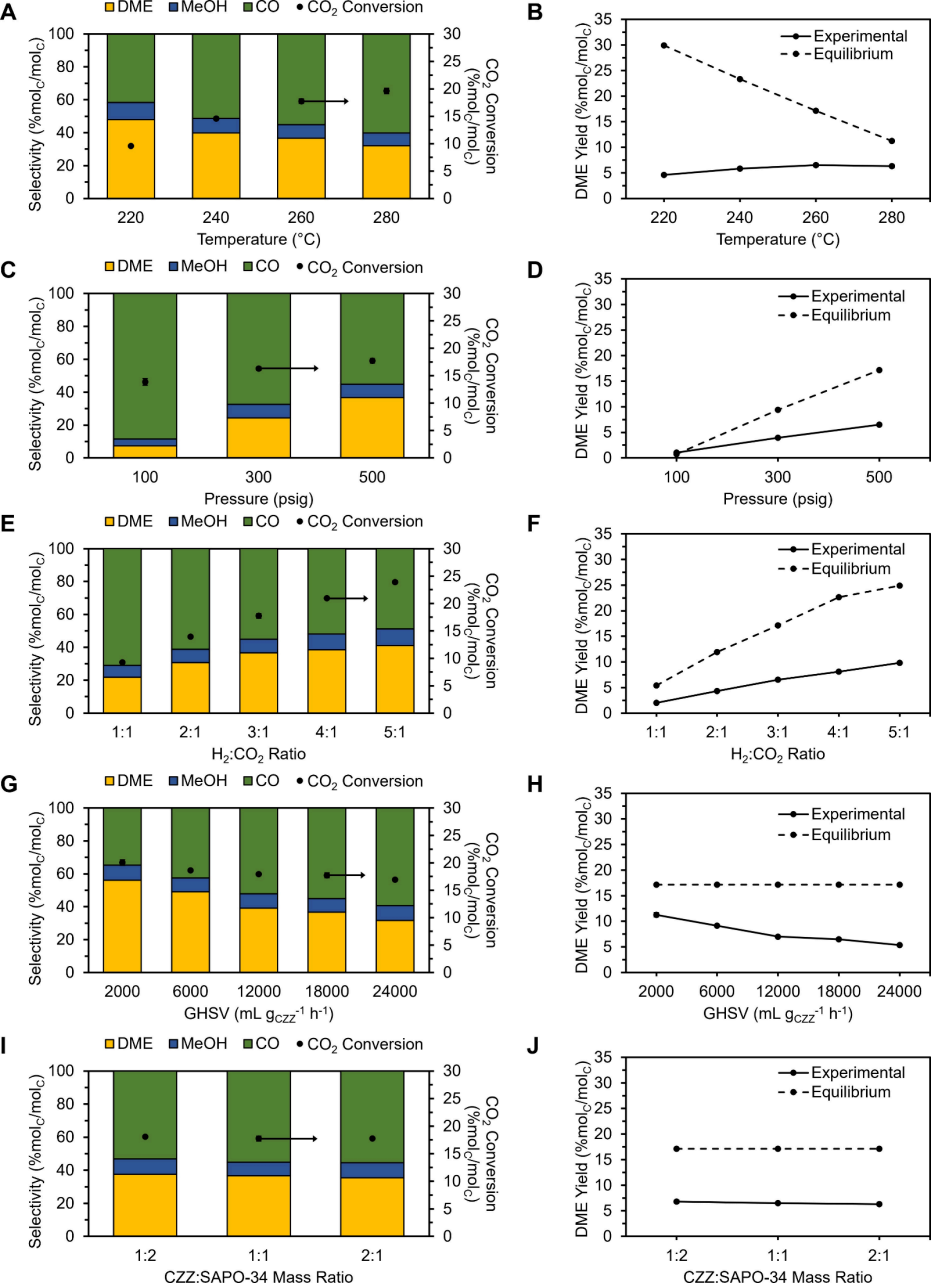
Catalytic performance of CZZ/SAPO-34 at varied A,B) temperature (220, 240, 260, 280 °C), C,D) pressure (100, 300, 500 psig), E,F) H_2_:CO_2_ ratio (1:1, 2:1, 3:1, 4:1, 5:1), G,H) GHSV (2000, 6000, 12000, 18000, 24000 mL g_CZZ_^–1^ h^–1^), and I,J) CZZ:SAPO-34 mass ratio (1:2, 1:1, 2:1). For A–I, the left axis shows product selectivity (% mol_C_/mol_C_) with yellow, blue, and green bars representing DME, MeOH, and CO, respectively. The right axis shows CO_2_ conversion (% mol_C_/mol_C_) represented by black circles. For B, D, F, H, and J, the solid line shows the DME yield from experimental data, while the dashed line shows the equilibrium DME yield. Base reaction conditions: 260 °C, 500 psig, 18000 mL g_CZZ_^–1^ h^–1^, H_2_:CO_2_ ratio = 3:1, mass of CZZ = 0.5 g, CZZ:SAPO-34 mass ratio = 1:1, GM configuration. Carbon balances were within ± 3% error.

Performing the reaction at an appropriate temperature is important to maximize the DME yield due to competition with the rWGS reaction. Looking at the effect of temperature (Figure 4A), increased CO_2_ conversions (∼10% at 220 °C and ∼20% at 280 °C) were observed at increasing temperatures although CO selectivity (∼42% at 220 °C and ∼60% at 280 °C) became favorable due to the endothermic nature of the rWGS reaction. In contrast, MeOH and DME selectivity dropped due to the exothermicity of the CO_2_ hydrogenation and MeOH dehydration reactions.^4,15,50^ Hence, the right balance among the temperature, CO_2_ conversion, and DME selectivity must be considered to obtain optimum DME yields. Results showed that a reaction temperature of 260 °C produced the most DME (Figure 4B) for the CZZ/SAPO-34 system under the base reaction conditions.

Higher CO_2_ conversion and DME selectivity were observed with increased reaction pressure (Figure 4C,D) and H_2_:CO_2_ ratio (Figure 4E,F) which agrees with the thermodynamics of CO_2_ to DME conversion (eqs 1–3).^15,54^ The overall CO_2_ to DME reaction involves 8 mol of reactant with only 4 mol of product indicating that high pressure favors DME formations per Le Châtelier’s principle. Similarly, the effect of H_2_:CO_2_ ratio can be explained by Le Châtelier’s principle. The stoichiometric ratio of H_2_:CO_2_ for the CO_2_ to DME conversions is 3:1. Thus, an H_2_-rich feed favors the formation of products, while an H_2_-lean feed supports the selectivity to CO.^15^

On the other hand, varying the gas-hourly space velocity (GHSV) (Figure 4G,H) resulted in an opposite trend compared to pressure and H_2_:CO_2_ ratio. As the GHSV was decreased, a rise in CO_2_ conversion (∼17% at 24000 mL g_CZZ_^–1^ h^–1^ to ∼20% at 2000 mL g_CZZ_^–1^ h^–1^) was observed with improved DME selectivity (∼32% at 24000 mL g_CZZ_^–1^ h^–1^ to ∼56% at 2000 mL g_CZZ_^–1^ h^–1^). Lower GHSV increases the residence time, allowing the system to reach its equilibrium concentrations and resulting in increased conversion and DME yield. Meanwhile, adjusting the mass ratio of CZZ:SAPO-34 resulted in minimal changes in the performance (Figure 4I) and DME yield (Figure 4J). This suggests that MeOH dehydration in SAPO-34 is likely fast and that MeOH formation controls the kinetics of the CO_2_ to DME reaction.

#### 3.2.3 Stability Test

We next studied the activity of CZZ/SAPO-34 for long time-on-stream (50 h) using optimized conditions from the parametric studies (260 °C, 500 psig, 2000 mL g_CZZ_^–1^ h^–1^, H_2_:CO_2_ ratio = 3:1, mass of CZZ = 0.5 g, CZZ:SAPO-34 mass ratio = 2:1, GM configuration) to investigate the stability of the catalyst, as shown in Figure 5. Minimal changes in the CO_2_ conversion (∼20%) and CO selectivity (∼34%) were observed for 50 h. DME selectivity decreased slightly from ∼60% to ∼56% after 14 h and remained consistent until 50 h. Consequently, MeOH selectivity increased from ∼4% to ∼10% after 9 h and stayed constant until 50 h. The decrease in DME and increase in MeOH selectivity point to a slight reduction in activity of SAPO-34 in the first few hours of operation. However, looking at the thermogravimetric analysis (TGA) of the spent SAPO-34 (Figure S9), there was no discernible difference when compared with the TGA of pretreated SAPO-34 indicating minimal coke formation, suggesting coke to not be the reason for the minor changes in reactivity. This shows that SAPO-34 has the potential to be a high performing and stable acid catalyst for CO_2_ to DME reactions.

**Figure 5:**
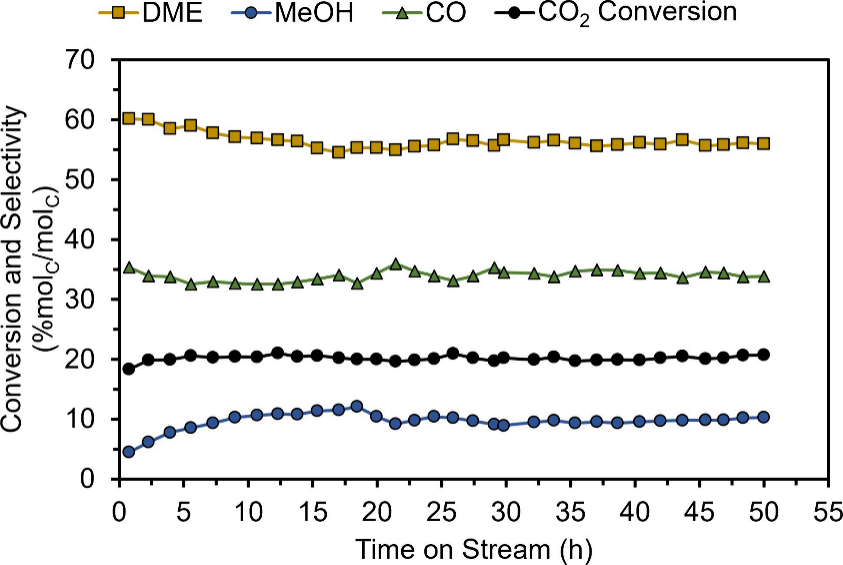
Performance of CZZ/SAPO-34 for the hydrogenation of CO_2_ to DME for 50 h of operation. Yellow squares, blue circles, and green triangles represent DME, MeOH, and CO selectivities (% mol_C_/mol_C_), respectively, while black circles show CO_2_ conversion (% mol_C_/mol_C_). Reaction conditions: 260 °C, 500 psig, 2000 mL g_CZZ_^–1^ h^–1^, H_2_:CO_2_ ratio = 3:1, mass of CZZ = 0.5 g, CZZ:SAPO-34 mass ratio = 2:1, GM configuration.

Overall, tests on the optimization of CZZ showed that CZZ-611 synthesized with an aging temperature of 40 °C and calcination temperature of 500 °C had the highest yield for MeOH. When coupled in a tandem configuration with SAPO-34, optimum DME yields can be attained by using a granular-mixed (GM) configuration operated at 260 °C, 500 psig, 2000 mL g_CZZ_^–1^ h^–1^, H_2_:CO_2_ ratio = 3:1, mass of CZZ = 0.5 g, and CZZ:SAPO-34 mass ratio = 2:1.

### 3.3 Kinetic Model Fitting

To develop a process model as the basis for a techno-economic analysis (TEA), a kinetic model would need to be established for the CZZ/SAPO GM system to account for changes in feed composition following the implementation of recycle streams in the model (*vide infra*). To do this, experimental data from the parametric studies were fitted against an existing kinetic model.^33^

The kinetic model and fitting procedure were adapted from Wild et al.^33^ who used a six-parameter model for MeOH synthesis and a three-parameter model for MeOH dehydration. Previous theoretical studies suggested that CO hydrogenation is insignificant at moderate or high CO_2_ pressures.^55,56^ Hence, only the reverse water–gas shift (rWGS) and CO_2_ hydrogenation reactions were considered for the MeOH synthesis model. The reaction rates for rWGS (*r*_1_, mol s^–1^) and CO_2_ hydrogenation (*r*_2_, mol s^–1^) are as follows:^33,57^

15 $$\begin{array}{c} r_{1}=m_{\mathrm{CZZ}}\cdot \exp\left(A_{1}-\frac{E_{A,1}}{RT}\right)\varphi_{\mathrm{Zn}}\theta_{b}\theta_{c}P_{{\mathrm{CO}}_{2}}P_{H_{2}O}\left(1-\frac{P_{\mathrm{CO}}P_{H_{2}O}}{P_{H_{2}}P_{{\mathrm{CO}}_{2}}K_{P,1}^{0}}\right) \end{array}$$

16 $$\begin{array}{c} r_{2}=m_{\mathrm{CZZ}}\cdot \exp\left(A_{2}-\frac{E_{A,2}}{RT}\right)\varphi_{\mathrm{Zn}}\theta_{b}\theta_{c}P_{H_{2}}^{1.5}P_{{\mathrm{CO}}_{2}}\left(1-\frac{P_{{\mathrm{CH}}_{3}\mathrm{OH}}P_{H_{2}O}}{P_{H_{2}}^{3}P_{{\mathrm{CO}}_{2}}K_{P,2}^{0}}\right) \end{array}$$

17 $$\begin{array}{c} \theta_{b}=\left(\frac{1}{1+K_{1}P_{{\mathrm{CO}}_{2}}P_{H_{2}}^{0.5}}\right) \end{array}$$

18 $$\begin{array}{c} \theta_{c}=\left(\frac{1}{1+K_{2}P_{H_{2}O}P_{H_{2}}^{-0.5}}\right) \end{array}$$

where *m*_CZZ_ is the mass of CZZ catalyst (kg), *A*_1_ and *A*_2_ are the pre-exponential factors, *E*_*A*,1_ and *E*_*A*,2_ are the activation energies (J/mol), φ_Zn_ is the coverage of zinc on the catalyst surface (0.1 for CO_2_/CO_*x*_ > 0.9),^33,57^ θ_b_ is the coverage of free Cu/Zn sites, θ_c_ is the coverage of free sites for H_2_O and H_2_, *P*_*i*_ is the pressure of gas species *i* (bar), *K*_1_ (bar^–1.5^) and *K*_2_ (bar^–0.5^) are the adsorption constants, and *K*_*P*,1_^0^ and *K*_*P*,2_^0^ are the reaction equilibrium constants which were computed based on the following equations:^57^

19 $$\begin{array}{c} K_{P,1}^{0}=T^{-1.097}\cdot \exp\left(-5337.4T^{-1}+12.569\right) \end{array}$$

20 $$\begin{array}{c} K_{P,2}^{0}=T^{-4.481}\cdot \exp\left(4755.7T^{-1}+8.369\right) \end{array}$$

The dehydration of MeOH was based on an associative reaction mechanism^58^ which has the following reaction rate:^33^

21 $$\begin{array}{c} r_{3}=m_{\mathrm{SAPO}34}\cdot \exp\left(A_{3}-\frac{E_{A,3}}{RT}\right)\theta_{d}P_{\mathrm{MeOH}}^{2}\left(1-\frac{P_{\mathrm{DME}}P_{H_{2}O}}{P_{\mathrm{MeOH}}^{2}K_{P,3}^{0}}\right) \end{array}$$

22 $$\begin{array}{c} \theta_{d}=\left(\frac{1}{1+K_{3}P_{\mathrm{MeOH}}}\right) \end{array}$$

where *m*_SAPO34_ is the mass of SAPO-34 (kg), *A*_3_ is the dehydration pre-exponential factor, *E*_A,3_ is the dehydration activation energy, θ_d_ is the coverage of free zeolite sites, *K*_3_ is the adsorption constant, and *K*_*P*,3_^0^ is the reaction equilibrium constant computed using eq 23.^59^

23 $$\begin{array}{c} K_{P,3}^{0}=0.106\cdot \exp\left(\frac{2.1858\times 10^{-4}}{RT}\right) \end{array}$$

The external and internal mass transfer limitations were assumed to be negligible. Experimental tests showed minimal mass transfer limitations (section S4).

From the rate equations, nine parameters were initially estimated: three pre-exponential factors (*A*_1_, *A*_2_, and *A*_3_), three activation energies (*E*_A,1_, *E*_A,2_, and *E*_A,3_), and three adsorption constants (*K*_1_, *K*_2_, and *K*_3_). Like Wild et al., *E*_A,3_ was found to be insignificant probably because MeOH dehydration is at quasi-equilibrium in the operating parameters tested. Hence, only 8 parameters were considered, and *E*_A,3_ was assumed to be zero.

The kinetic parameters were estimated by minimizing the objective function (χ^2^) as defined in eq 24.

24 $$\begin{array}{c} \chi^{2}=\overset{N_{P}}{\underset{j=1}{\sum }}{\left[\frac{{\left(X_{{\mathrm{CO}}_{2},\exp}-X_{{\mathrm{CO}}_{2},\mathrm{kin}}\right)}^{2}}{{\left(X_{{\mathrm{CO}}_{2},\exp}\right)}^{2}}+\frac{{\left(S_{\mathrm{CO},\exp}-S_{\mathrm{CO},\mathrm{kin}}\right)}^{2}}{{\left(S_{\mathrm{CO},\exp}\right)}^{2}}+\frac{{\left(S_{\mathrm{MeOH},\exp}-S_{\mathrm{MeOH},\mathrm{kin}}\right)}^{2}}{{\left(S_{\mathrm{MeOH},\exp}\right)}^{2}}+\frac{{\left(S_{\mathrm{DME},\exp}-S_{\mathrm{DME},\mathrm{kin}}\right)}^{2}}{{\left(S_{\mathrm{DME},\exp}\right)}^{2}}\right]}_{j} \end{array}$$

where *X*_CO_2_,exp_ is the CO_2_ conversion based on experimental data, *X*_CO_2_,kin_ is the CO_2_ conversion calculated from the kinetic model, *S*_*i*,exp_ is the selectivity of species *i* based on experimental data, *S*_*i*,kin_ is the selectivity of species *i* calculated from the kinetic model, and *N*_P_ is the number of experimental data points. Minimization was performed using MATLAB’s fminsearch function.

Twenty-six experimental results from the conditions in Table S6 were used as the training set for determining the kinetic parameters. Each experimental result has four points (CO_2_ conversion and selectivity of CO, MeOH, and DME) corresponding to 104 data points for modeling. Twenty-four of these data points were randomly chosen for validation, while the other 80 were used in training the model.

The resulting kinetic model fitted well with experimental data, as shown in Figure S10 with a χ^2^ of 9.8. The tabulated kinetic parameters are listed in Table 4. Most of the errors of the fitted model were associated with MeOH selectivity, as seen in the parity plot (Figure 6). The errors could be attributed to product streams with low MeOH concentrations as these streams had higher measurement inaccuracies due to a smaller gas chromatograph peak area. Nonetheless, the kinetic model was used to simulate data at high DME yields and low MeOH concentrations such that the deviations of MeOH from the kinetic model were deemed acceptable for the techno-economic analysis.

**Table 4:** Estimated Kinetic Parameters

Parameter	Unit	Estimated Value
*A*_1_	-	24.36
*A*_2_	-	16.92
*A*_3_	-	–0.27
*E*_A,1_	kJ/mol	84.44
*E*_A,2_	kJ/mol	76.93
*K*_1_	bar^–1.5^	4.98
*K*_2_	bar^–0.5^	376.69
*K*_3_	bar^–1^	1.57

**Figure 6:**
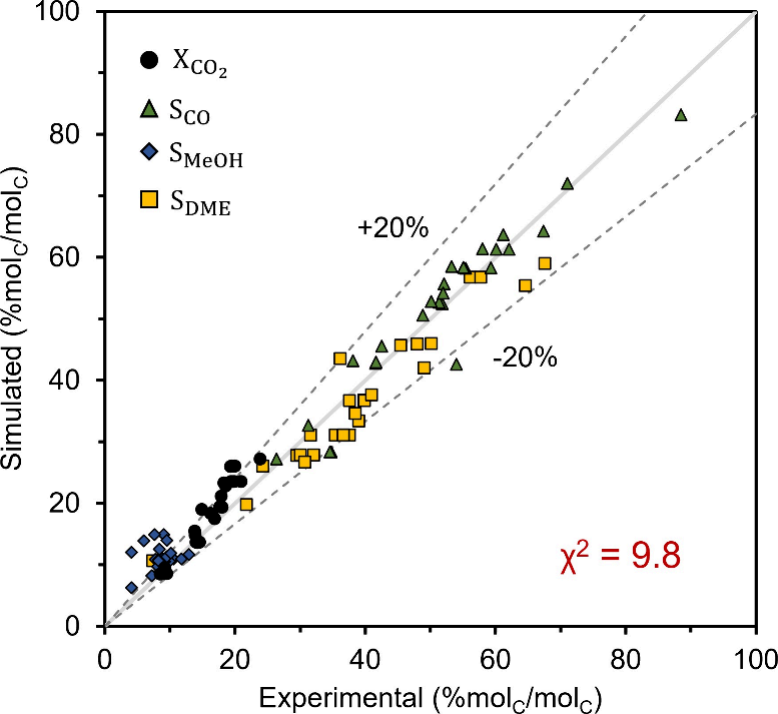
Parity plot comparing experimental data and simulated results from the fitted kinetic model. Yellow squares, blue circles, and green triangles represent DME, MeOH, and CO selectivity (% mol_C_/mol_C_), respectively, while black circles show CO_2_ conversion (% mol_C_/mol_C_). The dotted lines represent ±20% error.

### 3.4 Techno-economic Analysis (TEA)

#### 3.4.1 Technical Analysis

Using the fitted kinetic model and optimized reaction conditions from parametric studies, a process simulation for a CO_2_ to DME plant (process flow diagram, PFD in Figure 7) with 20,000 tons per year (tpy) capacity was designed in Aspen Plus v14. CO_2_ (S1) and H_2_ (S2) entered the plant and were compressed to 35 bar. These were then mixed with the unreacted gas recycle stream (S30), which was heated to 260 °C before going into the packed bed reactor (PBR1). The product gases then proceeded through a series of separation processes. The bulk of unreacted H_2_ and CO_2_ was separated through a flash drum (F1) in S14, which was recycled back to the reactor. Meanwhile, products such as MeOH, DME, and water went into S15, which was directed to three distillation columns for separating and purifying DME and MeOH. In the first column (DT1), unreacted gases, CO, and DME were recovered at the top (S18), while MeOH and water were collected at the bottom of the column (S19). S18 went to the second column (DT2), which purified DME (>99.9% purity) into S21, while the rest of unreacted gases and CO were recycled back to the reactor (S20). In parallel, S19 was directed to the third column, which separated and purified MeOH (>99.5% purity) at the top of the column (S23) and water (>99.4% purity) at the bottom (S24).

**Figure 7:**
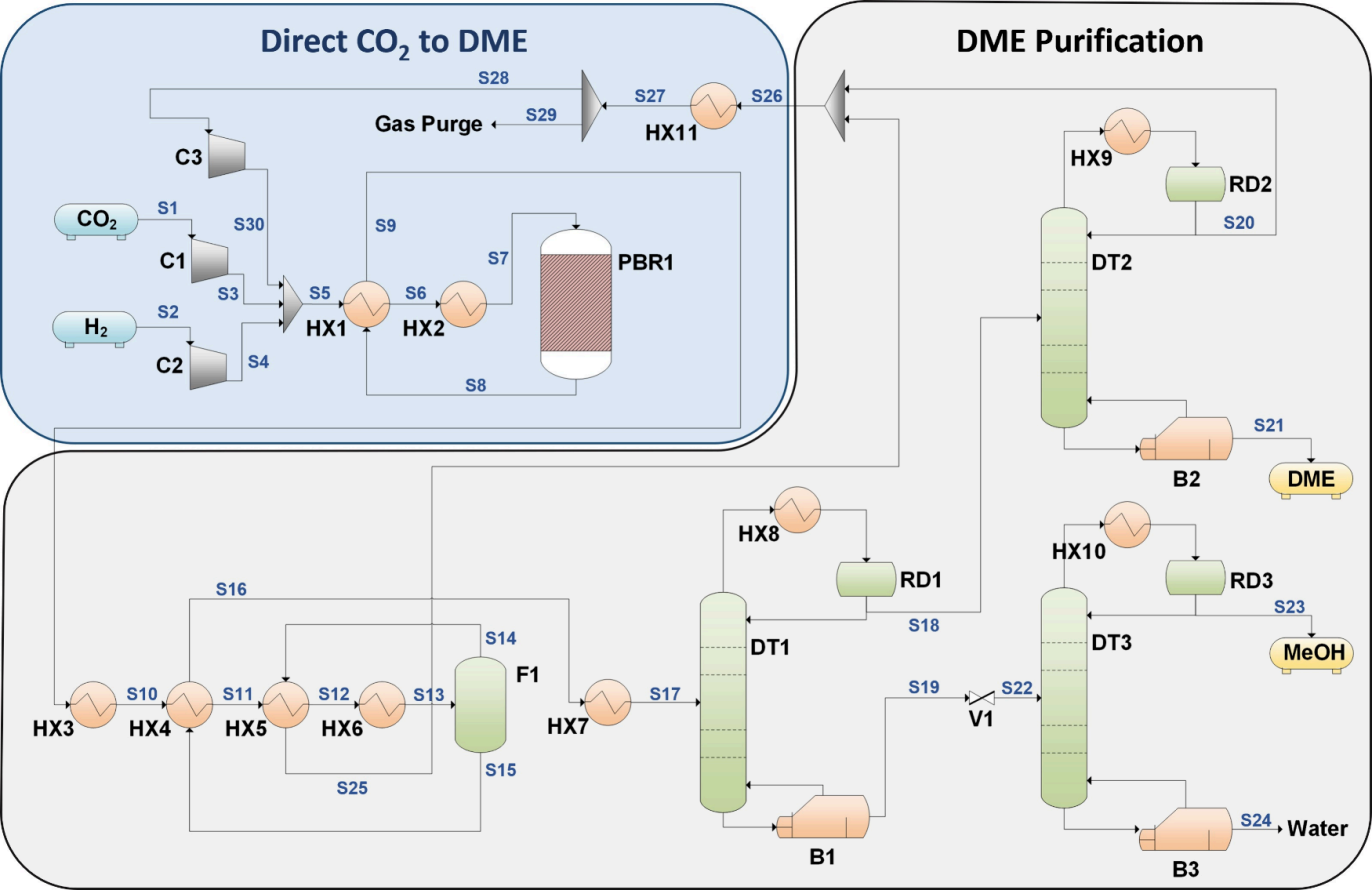
Process flow diagram (PFD) for the one-step hydrogenation of CO_2_ to DME. Utilities streams are omitted.

A summary of the technical performance indicators can be found in Table 5. The single-pass CO_2_ conversion (20.4%) and DME selectivity (55.3%) of the process model were similar to those observed in the laboratory-scale stability test performed for CZZ/SAPO-34 (*X*_CO2_ = 20% and *S*_DME_ = 56%) operated under the same conditions (see Section 3.2.3). This validated the successful incorporation of the experimental data into the process model through the developed kinetic model. Recycling the unreacted gases resulted in an overall CO_2_ conversion of 96.2% and a DME selectivity of 75.2%, which was close to the equilibrium distribution between DME (78.4%) and methanol (21.6%). The higher overall selectivity of DME compared to the per-pass selectivity was likely due to the recycling of CO and its further conversion into MeOH and DME. At a steady state, the recycle-to-feed ratio was 4.0 for H_2_ and 3.7 for CO_2_. A conversion factor of 0.4 was obtained, which indicates that for every 1 kg of CO_2_ fed into the plant, 0.4 kg of DME was generated (76.5% of theoretical maximum yield).

**Table 5:** Performance Indicators of the Designed CO_2_ to Dimethyl Ether (DME) Plant

Performance Indicator	Value	Unit
Per pass CO_2_ conversion	20.4	% mol_C_/mol_C_
Per pass DME selectivity	55.3	% mol_C_/mol_C_
Overall CO_2_ conversion	96.2	% mol_C_/mol_C_
Overall DME selectivity	75.2	% mol_C_/mol_C_
H_2_ feed	7.6	kton/y
CO_2_ feed	55.3	kton/y
H_2_ recycle-to-feed ratio	4.0	kton/kton
CO_2_ recycle-to-feed ratio	3.7	kton/kton
DME productivity	20.0	kton/y
Conversion factor	0.4	kton DME/kton CO_2_

#### 3.4.2 Economic Analysis

From the process simulation in Aspen Plus v14, an economic analysis was performed using the Aspen Process Economic Analyzer (APEA). The total estimated purchased equipment cost (PEC) amounted to $13.7 M with the distribution shown in Figure 8A. A major portion was associated with the cost of compressors (58%) followed by the packed bed reactor (24%). Heat exchangers and distillation towers corresponded to 5% and 3% of PEC, respectively. Flash drums, pumps, and turbines only accounted for ∼1% of the PEC. Lastly, equipment (boiler and cooling tower) for generation of utilities contributed 9% of the PEC. A total CAPEX of $81.5 M was estimated for a 20,000 tpy capacity CO_2_ to DME plant. The complete components of CAPEX can be found in Table S9.

**Figure 8:**
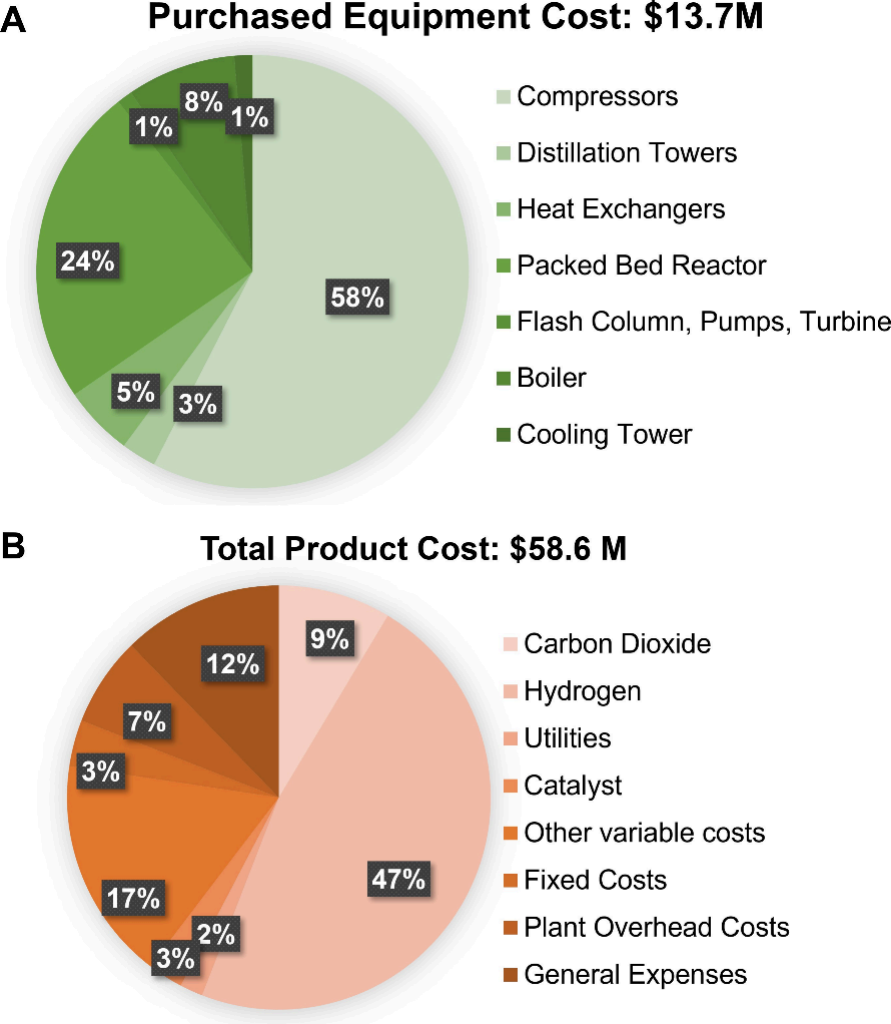
A) Breakdown of purchased equipment cost (PEC) obtained from Aspen Plus v14’s Aspen Process Economic Analyzer (APEA). B) Breakdown of operational expenditure (OPEX) based on raw material, utility, and catalyst usage and other variable and fixed costs estimated based on OPEX factors.^40^

An annual operational expenditure (OPEX) of $58.6 M was estimated for a 20,000 tpy capacity of CO_2_ to DME plant. The breakdown of the OPEX was depicted in Figure 8B. A significant portion of the OPEX came from the cost of H_2_ (47%), while CO_2_ only accounted for 9%. Utilities and catalysts constituted 2% and 3% of the OPEX, respectively. The rest of the OPEX is comprised of other variable costs (i.e., operating labor and supervision, operating supplies, laboratory charges, royalties), fixed costs (i.e., taxes, insurance), plant overhead costs, and general expenses (i.e., administrative costs, distribution and marketing, research and development) estimated through OPEX factors.^40^

To better understand the economics of the conversion of CO_2_ to DME processes, we computed the annual DME production cost (ADPC) and minimum DME selling price (MDSP) as summarized in Table 6. The ADPC amounted to $66.6 M and was mostly dominated by the OPEX (∼88%) with CAPEX constituting only a small portion (∼12%). The calculated MDSP was $3.21/kg. Comparing it with the current value of DME which is $1.2/kg,^60^ DME from CO_2_ was roughly 2.7 times more expensive. Even so, it is best to note that the process comes with decreased CO_2_ emissions, which is important to consider in the future, especially with the worsening effects of global warming. In addition, the production cost of DME was mostly from operation costs, wherein a near majority comes from the supply of H_2_. This signifies the importance of lowering H_2_ prices in decreasing the production cost of DME from CO_2_.

**Table 6:** Economic Indicators of the Designed CO_2_ to Dimethyl Ether (DME) Plant

Economic indicators	Value	Unit
CAPEX	81.5	$M
Annualized CAPEX (CAPEX_Annual_)	8.0	$M
OPEX	58.6	$M
Annual DME Production Costs (ADPC)	66.6	$M
Minimum DME Selling Price (MDSP)	3.21	$ kg^–1^

Based on the MDSP, DME from CO_2_ hydrogenation is not yet economically competitive with DME from natural gas. However, commodity prices fluctuate over time and vary significantly depending on the source or industry from which they are obtained. Hence, a sensitivity analysis on MDSP (Figure 9) was performed to get insights into how these changes affect the cost of DME. We also investigated the effect of changes in CAPEX, tax and interest rates, and catalyst lifetime.

**Figure 9:**
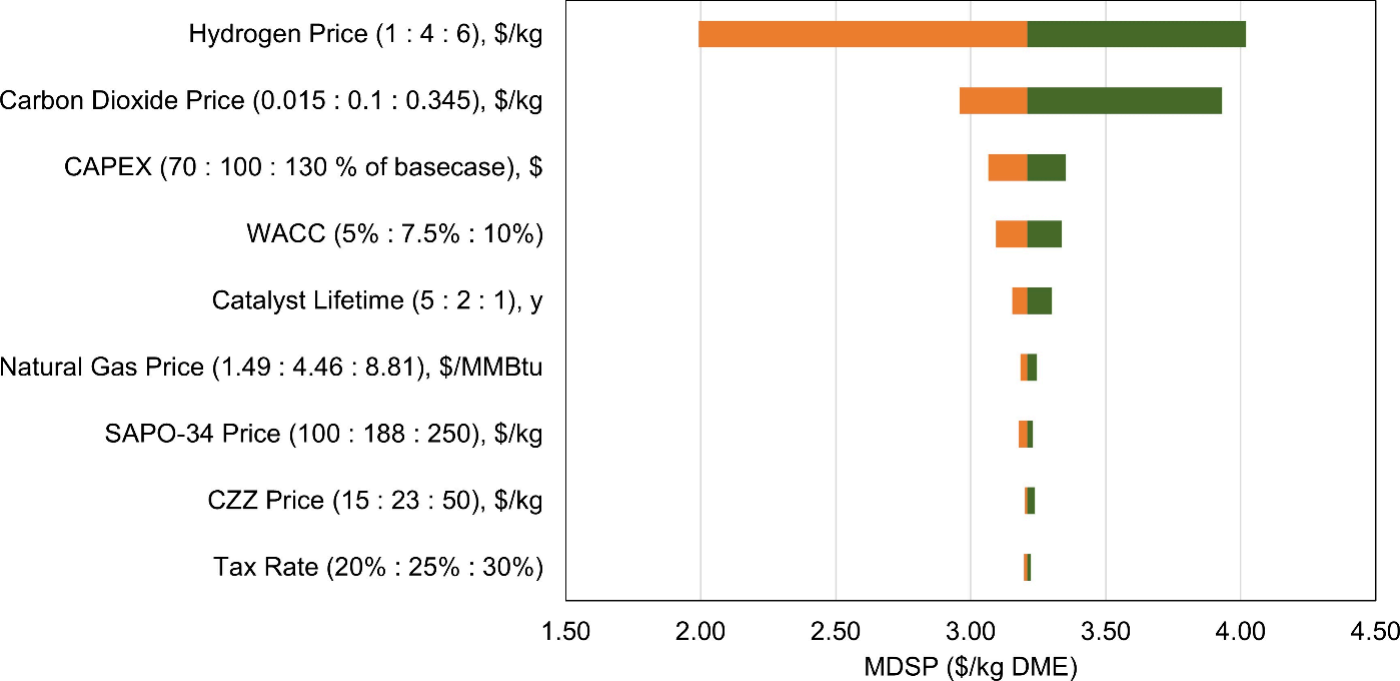
Effect of the different cost factors on the minimum DME selling price (MDSP) of the direct hydrogenation of CO_2_ to DME.

The cost of hydrogen had the most impact on MDSP. Even with the use of the cheapest hydrogen from natural gas (gray hydrogen, $1–2/kg^41,61^ ), the MDSP only lowers to ∼$1.99/kg, still higher than the current value of DME ($1.2/kg^60^). The cost of CO_2_ also had notable effects on MDSP. The price of captured CO_2_ can vary among industries and is affected by factors such as the volume, CO_2_ concentration, and pressure of exhaust gases in a plant.^62^ Utilization of captured CO_2_ from natural gas processing, which has a cost of $0.015–0.025/kg CO_2_,^63^ could reduce the MDSP to as low as ∼ $2.96/kg while the use of the more expensive CO_2_ directly captured from air ($0.135–0.345/kg CO_2_)^62^ could result in MDSP as high as ∼$3.93/kg. The CAPEX, interest rates, and catalyst lifetimes were observed to substantially affect the MDSP, resulting in a change ranging from ∼$3.07/kg to ∼$3.35/kg. Utility cost, catalyst cost, and tax rate had a small impact on MDSP. Overall, the sensitivity analysis highlights that the economics of CO_2_ hydrogenation to DME is mainly driven by hydrogen costs, further emphasizing the importance of reducing green hydrogen costs.

### 3.5 Carbon Footprint

The CO_2_ to DME plant is desired to have zero or negative overall CO_2_ emission. Thus, we looked into the cradle-to-gate carbon footprint (kg CO_2_ eq/kg DME) of the CO_2_ to DME plant and how it changes with varying H_2_, CO_2_, and boiler energy sources as depicted in Figure 10. The cradle-to-gate analysis covers emissions from the acquisition of raw materials until the point it leaves the CO_2_ to the DME plant gate. As a base case, H_2_ was assumed to come from electrolyzers powered by renewable energy (0 kg CO_2_ eq/kg DME), CO_2_ was captured from an external industrial plant (0 kg CO_2_ eq/kg DME), and the steam boiler was powered by natural gas (0.13 kg CO_2_ eq/kg DME). CO_2_ captured from an external plant was considered to have an emission of zero as it has not entered or come from the atmosphere. At these assumptions, the total CO_2_ footprint of the base case is 0.21 kg of CO_2_ eq/kg of DME, which includes the emitted greenhouse gases (GHGs) from the process purge line having an equivalent emission of 0.08 kg of CO_2_ eq/kg of DME.

**Figure 10:**
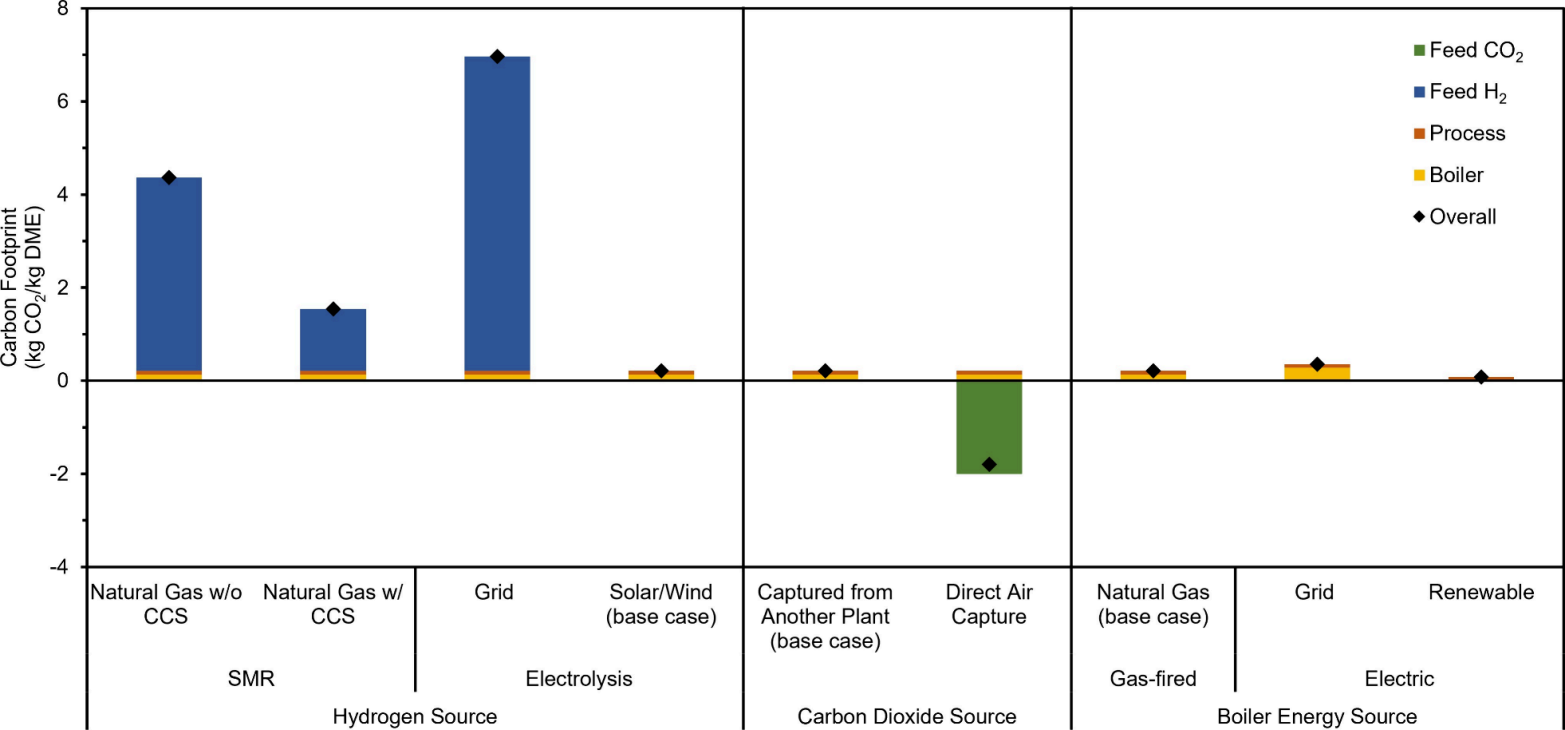
Effect of varying the H_2_ source, the CO_2_ source, and the boiler energy source on the carbon footprint of the CO_2_ to DME plant. Green, blue, orange, and yellow bars represent equivalent CO_2_ emissions from feed CO_2_, feed H_2_, process emissions, and steam boiler, respectively, while the black diamond shows the overall CO_2_ emissions.

Among the three variables investigated, H_2_ had the most impact on the overall carbon footprint of the CO_2_ to DME process. Steam methane reforming (SMR) of natural gas for H_2_ production (gray H_2_) releases significant amounts of CO_2_ (12 kg CO_2_ eq/kg H_2_).^41^ When used as a hydrogen source, the total footprint rose to 4.4 kg CO_2_ eq/kg DME. However, when SMR is coupled with CCS at 93% capture rate (blue H_2_), the carbon footprint can be significantly reduced to 1.5 kg CO_2_ eq/kg DME. Surprisingly, utilizing hydrogen from electrolyzers that rely on electricity from the grid led to a higher carbon footprint (7.0 kg CO_2_ eq/kg DME) due to the high electricity demand of current electrolyzers and the fact that a major portion (∼60%) of electricity from the grid comes from natural gas and coal.^64^ On the other hand, changing the CO_2_ source to direct air capture (DAC) resulted in a negative carbon footprint of −1.8 kg CO_2_ eq/kg DME as CO_2_ is directly removed from air and converted to DME. However, it is important to note that these are only cradle-to-gate emissions. When considering DME consumption, GHG emissions should be captured and prevented from entering the atmosphere to maintain negative emissions on a cradle-to-grave basis. Going back to the base case, a significant contributor to carbon emissions comes from the steam boiler. Using electric boilers powered by the grid resulted in higher footprint (0.36 kg CO_2_ eq/kg DME) relative to the base case while using renewable energy led to a minimal emission of 0.08 kg CO_2_ eq/kg DME.

## 4 Discussion

To summarize, we determined the aging temperature (40 °C), calcination temperature (500 °C), and composition of CZZ (611) that gave the highest MeOH yield. We then looked into the performance of CZZ/SAPO-34 and optimized its conditions (260 °C, 500 psig, 2000 mL g_CZZ_^–1^ h^–1^, H_2_:CO_2_ ratio = 3:1, mass of CZZ = 0.5 g, and CZZ:SAPO-34 mass ratio = 2:1, GM configuration) to favor DME yield. Through the development of kinetic and process models, we evaluated the economics and environmental footprint of the process, considering a DME plant capacity of 20,000 tpy, wherein we identified the feed hydrogen source to significantly affect the economics and cradle-to-gate carbon footprint of the process.

From the catalyst evaluation studies, the investigation on the aging and calcination temperatures has revealed that aging at 40 °C and calcination at 500 °C resulted in the CZZ structure with the highest MeOH yield. Several studies on CZZ performed aging at 80–90 °C and calcination at 300–400 °C but did not report whether these conditions were optimized or adapted from a previous study.^28,29,31,65^ This work points out the importance of performing synthesis optimization to generate results that fully exhibit the potential of the catalyst synthesized. In terms of composition, CZZ-611 had the highest activity, which is likely due to the increased number of Cu sites, generally considered as the active phase of the catalyst,^66^ and higher Cu/ZnO and/or Cu/ZrO_2_ interfaces and higher activity. Our results suggest that Cu/metal oxide interfaces are critical to the hydrogenation of CO_2_ as Cu on its own is not efficient in producing MeOH. The direct contact between Cu metal and the basic sites of ZnO may favor the formation of Cu/Zn alloys, which have been shown to provide dual binding sites for the activation of CO_2_ and catalyze its hydrogenation.^67,68^ Meanwhile, ZrO_2_ has been suggested to interact with metallic Cu, which creates active sites for the selective reaction to MeOH and helps in increasing MeOH selectivity.^49,69^ The combination of these synergetic effects likely resulted in a high MeOH yield in this study. We note that the optimum composition of CZZ (71 wt % Cu and 12 wt % Zn) determined in this work is close to the commonly used composition of CZA used industrially (50–70% wt % Cu and 20–50 wt % Zn).^22,48,70,71^

We then paired the optimized CZZ-611 with SAPO-34 and showed the capability of SAPO-34 to be utilized in the direct hydrogenation of CO_2_ to DME. Different reactivities at different proximities of the CZZ and SAPO-34 active sites demonstrated the importance of a proper bed configuration in tandem systems. In a dual-bed configuration, the reaction in the top bed (CO_2_ to MeOH) proceeds independently from the second bed (*akin* to a single-bed configuration consisting of only a CZZ catalyst). The products of the CO_2_ to MeOH reaction leave the top bed and reach SAPO-34 where the MeOH produced is converted to DME. Thus, the reaction happens in sequence in the dual-bed configuration. Mixing CZZ with SAPO-34 in a GM, PM, or MM configuration creates an environment wherein the CO_2_ to MeOH reaction occurs simultaneously with the MeOH to DME reaction since the metal oxide and acidic active sites are in close proximity. In this case, MeOH (produced from CZZ) is immediately consumed in SAPO-34 which effectively lowers instantaneous concentrations of MeOH in the mixed catalyst bed. By Le Châtelier’s principle, this pushes the CO_2_ to MeOH reaction to proceed further, thus producing more MeOH which eventually converts to DME in SAPO-34. Thus, the presence of both active sites in the bed hastens the consumption of the MeOH intermediate due to SAPO-34 and pushes CZZ to produce more MeOH which overall increases the total MeOH (MeOH + DME) formed, as observed in the experiments conducted. However, no improvements were observed between GM and PM/MM, which denotes that the reaction is not mass transfer limited but rather kinetically controlled. In all tests, SAPO-34 effectively converted MeOH to DME at the temperature range tested (220–280 °C), reaching near equilibrium distributions between MeOH and DME. In addition, SAPO-34 showed 100% selectivity to DME and displayed exceptional stability and no signs of deactivation for long hours (50 h) of operation, demonstrating its excellent applicability in CO_2_ to DME conversions. We acknowledge that the optimum operating conditions identified in this study (260 °C, 500 psig, 2000 mL g_CZZ_^–1^ h^–1^, H_2_:CO_2_ ratio = 3:1, mass of CZZ = 0.5 g, CZZ:SAPO-34 mass ratio = 2:1) are restricted by operational limitations in the laboratory. Higher CO_2_ conversions and DME selectivity could ideally be further achieved by performing the reaction at higher pressures and lower GHSV.

Economic analysis of a 20,000 tpy DME plant showed that the industrial feasibility of hydrogenation of CO_2_ to DME is highly reliant on raw material prices, especially H_2_. Assuming the use of green H_2_ and captured CO_2_ from industrial plants, an MDSP of $3.21/kg DME was identified and has minimal cradle-to-gate carbon footprint (0.21 kg CO_2_ eq/kg DME). A lower MDSP of $1.99/kg DME was estimated when using H_2_ sourced from steam methane reforming. However, this comes at the cost of a high carbon footprint (4.4 kg of CO_2_ eq/kg of DME). Clearly, there is an inverse relationship between the cost of producing DME and a carbon footprint. Low DME production costs with low CO_2_ emissions rely on the development of cheaper green H_2_ technologies. According to a study by Detz et al.,^72^ green H_2_ costs will be competitive with gray H_2_ between 2025 and 2048 in an optimistic scenario. However, current green H_2_ costs still range from $3.5/kg H_2_ to $5/kg H_2_,^41^ signifying that it will not be competitive with gray H_2_ anytime soon. Even when assuming low H_2_ ($1/kg H_2_) and CO_2_ ($0.015/kg H_2_) prices, MDSP only lowers to ∼$1.74/kg DME which is still ∼1.5× more expensive than current DME prices ($1.2/kg^60^). Hence, further development of CO_2_ hydrogenation technologies and increased catalyst efficiencies are still critical in making the technology competitive.

To show the effect of improving reactor and catalyst activity, we performed a comparative analysis, focusing only on the reaction section of the plant (Figure S11). We compared a two-reactor model (indirect), wherein one reactor contained CZZ while the other has SAPO-34, and a single-reactor model (direct), wherein granular mixtures of the two catalysts are present. Details of this analysis can be seen in Section S8 of the SI. The single-reactor model had a higher per pass CO_2_ conversion and per pass DME selectivity compared to the two-reactor model, which is expected, as demonstrated by the tandem studies (GM vs DB). The higher activity of the single-reactor model resulted in a smaller recycling stream (as indicated by the lower H_2_ and CO_2_ recycle-to-feed ratio) and hence had smaller equipment and heating requirements. The savings on reactor cost alone was equivalent to ∼46% when switching from a two-reactor to a single-reactor. There was also an ∼32% reduction in heat exchanger PEC due to the smaller size requirements caused by the smaller recycling stream. Considering that ∼24% of the overall PEC (Figure 8A) was attributed to the packed bed reactor, then this translates to significant savings in the overall CAPEX. Furthermore, the ∼33% lower heat duty with the single-reactor means ∼33% lower utilities consumption, specifically natural gas, and with it comes ∼33% reduction in CO_2_ emissions from steam generation. Although this does not considerably affect OPEX as utilities only comprised 2% (Figure 8B), it is, however, significant in terms of plant emissions. In the base case scenario of our carbon footprint analysis, ∼61% of the overall plant emissions came from steam generation; therefore, any reduction in equivalent CO_2_ emitted from natural gas would be significant. As we can see in this analysis, a decrease in CAPEX and CO_2_ emissions can be achieved by increasing the efficiency of the process through either a better reactor design or more active catalysts. Even though MDSP mostly accounts for OPEX which is currently dominated by H_2_ costs, the increasing global efforts in making green H_2_ cheaper could mean that the reduction of CAPEX and CO_2_ emissions (through improvements in process efficiencies) may soon play a larger role in the viability of the CO_2_ to DME technology.

## 5 Conclusion

In this work, a multipronged approach was taken to study CO_2_ hydrogenation to DME using tandem CZZ/SAPO-34 catalysts, from optimizing the catalytic performance to an assessment of its economic viability and environmental impact resulting from catalytic advances. CZZ-611 synthesized at an aging temperature of 40 °C and calcination temperature of 500 °C resulted in the highest activity for CO_2_ to MeOH reaction, reaching CO_2_ conversion of 13.7% and MeOH selectivity of 39.2% at 260 °C, 500 psig, and 18000 mL g_CZZ_^–1^ h^–1^. We then coupled CZZ with SAPO-34 in a tandem catalytic system, which effectively performed CO_2_ hydrogenation to DME. We observed that the presence (e.g., GM, PM, and MM configuration) of both active sites in the same bed improved the CO_2_ conversion and DME selectivity. In addition, high DME yields can be obtained at a temperature of 260 °C, high pressures (e.g., 500 and 700 psig), low GHSV (e.g., 2000 mL g_CZZ_^–1^ h^–1^), and high H_2_:CO_2_ ratios (e.g., 3:1, 4:1, and 5:1). On the other hand, varying CZZ:SAPO-34 mass ratios from 1:2 to 2:1 did not show significant changes in activity. The CZZ/SAPO-34 tandem system shows good stability for 50 h with a CO_2_ conversion of 20% and a DME selectivity of 56%.

An experimental data-fitted kinetic model was developed for the CZZ/SAPO-34 tandem system and applied to a process simulation to perform a techno-economic analysis (TEA) on a 20,000 tpy capacity plant. TEA revealed that the DME production cost was highly dependent on feedstock cost (H_2_ and CO_2_) rather than catalytic or operational variables. A minimum DME selling price (MDSP) of $3.21/kg was computed for the base case, which could be reduced to $1.99/kg if H_2_ costs were lowered to $1/kg. The CO_2_ to DME plant had a carbon footprint of 0.21 kg of CO_2_ eq/kg of DME, for the base case, which is greatly influenced by the type of hydrogen used. A negative CO_2_ emission of −1.6 kg CO_2_ eq/kg DME could be achieved when using CO_2_ captured from air. Using CO_2_ captured from other processes or plants results in only a nearly carbon-neutral DME production process. In sum, the economic and environmental impacts of a CO_2_ to DME plant are largely driven by the feedstock, hydrogen, and are, therefore, reliant on the development of cheaper green hydrogen technologies to make DME from CO_2_ more cost-effective and sustainable. Overall, this work gave a holistic view of the challenges of CO_2_ hydrogenation from catalyst design and synthesis up to scale-up and industrial operation.

## Supplementary Material



## Notes

Economic and carbon footprint analysis assumptions, aging and calcination studies, PXRD patterns, H_2_-TPR profiles, CO_2_-TPD profiles, additional reactivity, test on mass transfer limitation, thermogravimetric analysis, kinetic model data, detailed process model description and process stream data, OPEX estimation methods, and single-reactor vs two-reactor model comparison (PDF)
