# Supplementary material for: Tandem Cu/ZnO/ZrO2‑SAPO-34 System for Dimethyl Ether Synthesis from CO2 and H2: Catalyst Optimization, Techno-Economic, and Carbon-Footprint Analyses
Source: ACS Eng Au. 2025 Apr 8;5(3):267–83. doi: 10.1021/acsengineeringau.5c00008 (PMC12183728; doi:10.1021/acsengineeringau.5c00008)
Supplement: Supplementary file 1 [file eg5c00008_si_001.pdf]

Supporting Information

Tandem Cu/ZnO/ZrO_2-SAPO-34 System for Dimethyl Ether Synthesis from CO_2 and H_2: Catalyst Optimization, Techno-Economic and Carbon-Footprint Analyses

Jasan Robey Mangalindan[1], Fatima Mahnaz[1], Jenna Vito[1], Navaporn Suphavilai[1], & Manish Shetty[1,]*

[1]Artie McFerrin Department of Chemical Engineering, 100 Spence Street, Texas A&M University, College Station, TX 77843, USA.

*Email. manish.shetty@tamu.edu

S1. Assumptions on Economic and Carbon Footprint Analysis

Table S1. Lang factors used in computing CAPEX.[1]

	**Lang Factor**
Direct Costs (DC)	
Purchased equipment cost	1
Purchased equipment installation	0.47
Instrumentation and controls	0.36
Piping	0.68
Electrical system	0.11
Building (including services)	0.18
Yard Improvements	0.1
Service facilities	0.7
Total direct costs	
Indirect Costs (IC)	
Engineering and supervision	0.33
Construction expense	0.41
Legal expense	0.04
Contractor's fee	0.22
Contingency	0.44
Total indirect costs	
**Fixed Capital Investment (FCI)**	5.04
**Working Capital (WC)**	0.89
**CAPEX**	5.93

Table S2. Methodology for computing OPEX.[1]

	**Calculation Method**
Variable Costs	
Operating Labor (OL)	See section 7.2.3 in SI
Operating Supervision (OS)	0.15 of OL
Maintenance and Repairs (MR)	0.07 of FCI
Operating Supplies	0.15 of MR
Laboratory Charges	0.15 of OL
Royalties	0.04 of TPC
Fixed costs	
Taxes	0.02 of FCI
Insurance	0.01 of FCI
Plant Overhead Costs	0.6 of (OL + OS + MR)
General Expenses	
Administrative	0.2 of (OL + OS + MR)
Distribution and Marketing	0.05 of TPC
Research and Development	0.05 of TPC

Table S3. Equivalent CO_2 emissions assumed in the cradle-to-gate carbon footprint analysis.

Processes	Unit	Equivalent CO_2 Emitted (kg CO_2 eq/unit)	Reference
Hydrogen Production			
Steam methane reforming (SMR)	kg H_2	12	2
SMR with 93% carbon capture	kg H_2	3.85	2
Electrolysis – using grid	kg H_2	19.5[a]	2, 3
Electrolysis – using Solar/Wind	kg H_2	0	2
Carbon Dioxide Source			
External plant	kg CO_2	0[b]	-
Direct air capture (DAC)	kg CO_2	-1[c]	-
Boiler Energy Source			
Natural gas	MMBtu	52.91	4
Electricity – grid[d]	kWh	0.39	3
Electricity – Solar/Wind	kWh	0[e]	-

[a] Considering an electricity consumption of 50 kWh/kg H_2 and a CO_2 emission of 0.39 kg CO_2 eq/kWh.
[b] CO_2 captured from an external plant was considered to have 0 kg CO_2 eq emissions as the CO_2 has not entered the atmosphere yet and it had no effect on the CO_2 levels in the atmosphere.
[c] CO_2 captured from air was assumed equivalent to -1 kg CO_2 eq since CO_2 was removed from the atmosphere. DAC is assumed to be powered by renewable energy (solar/wind).
[d] Electricity consumed in electric boiler was assumed to be equal to the energy content of natural gas consumed from the process model using a conversion factor of 3412 Btu/kWh.
[e] Electricity from renewable sources such as solar/wind were assumed to have negligible CO_2 emissions.

S2. Effect of Aging and Calcination Temperatures on CZZ Activity

Aging tests were done at 40°C and 80°C followed by calcination at 500°C using an initial Cu:Zn:Zr molar ratio of 6:1:3 (CZZ-613). Different calcination temperatures (350°C and 600°C) were also performed for CZZ-613 aged at 40°C. The PXRD of the calcined CZZ-613 is shown in **Figure S1**. Catalysts calcined at 350°C and 500°C showed diffraction peaks at 2θ values of 32.4°, 35.6°, 38.8°, and 48.8° which respectively corresponds to (110), (002), (111), and (202) planes of CuO[5, 6] while no peaks of ZnO and ZrO_2 were observed indicating that these are in the amorphous phase or exists as highly-dispersed small crystals. Increasing the aging temperature from 40°C to 80°C and calcination temperature from 300°C to 500°C resulted in larger CuO

crystallite sizes as summarized in **Table S4**, which were computed by the Scherrer equation based on the (111) plane. Further raising the calcination temperature to 600°C resulted to stronger CuO peaks and the appearance of (002) and (101) planes of ZnO at 2θ = 34.4°, and 36.2°, respectively, along with *t*-ZrO_2 at 2θ = 30.4° and *m*-ZrO2 at 2θ = 28.3° and 31.5° signifying the formation of large crystallites which could indicate sintering of the metal oxide.

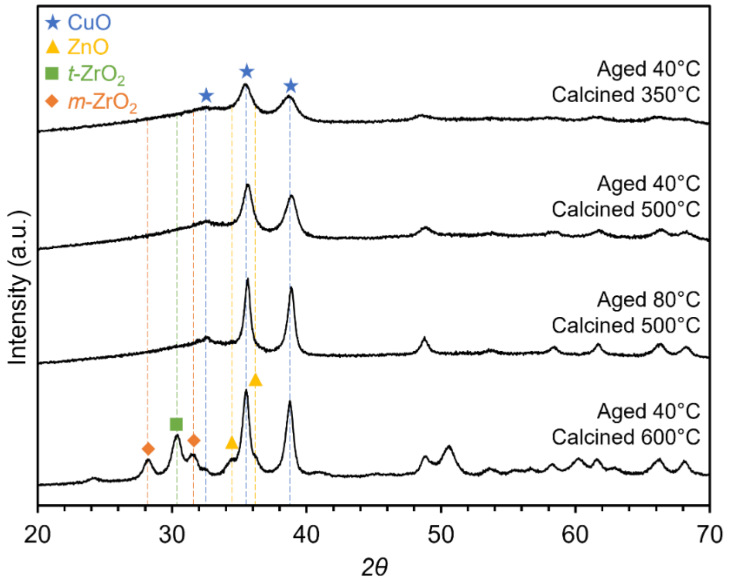

Figure S1. Powder x-ray diffraction (PXRD) pattern of calcined CZZ-613 produced with different aging and calcination temperatures.

Table S4 shows the reactivity of CZZ-613 for CO_2 hydrogenation to MeOH at 240°C, 500 psig, and 18,000 mL g_{CZZ}^{-1} h^{-1}. Results for reaction temperatures of 220°C, 260°C, and 280°C are also available in **Figure S2**. A rise in the aging temperature from 40°C to 80°C gave a lower CO_2 conversion (12% to 9%) and MeOH selectivity (37.2% to 35.5%). The larger crystallite sizes of CuO at 80°C suggests agglomeration of Cu atoms which translates to a decrease in Cu surface area. Previous studies have reported that CZZ reactivity is directly related to the Cu surface area which explains the decreased activity of the catalyst aged at 80°C.[7] On the other hand, increasing

the calcination temperature from 350°C to 500°C resulted in a nearly constant CO_2 conversion (~12%) with higher selectivity to MeOH (33.6% to 35.5%). The lower activity of the catalyst calcined at 350°C is hypothesized to be caused by the incomplete decomposition of the metal carbonates. To probe this, TGA of the dried CZZ-613 was performed as shown in **Figure S3A**. The bulk of the decomposition of CZZ occurred at 350°C but an appreciable change in weight (**Figure S3B**) was still observed up to 500°C, confirming that decomposition to metal oxide was incomplete at 350°C. Further raising the calcination temperature from 500°C to 600°C decreased the CO_2 conversion (12.0% to 10.5%) which can be attributed to sintering resulting in the loss of active sites. The MeOH yield was found to be optimum at an aging and calcination temperature of 40°C and 500°C, respectively.

Table S4. Catalytic activity (240°C, 500 psig, 18000 mL g_{CZZ}^{-1} h^{-1}) of CZZ-613 with different aging and calcination temperatures.

Aging Temperature (°C)	Calcination Temperature (°C)	CO_2 Conversion (%mol_C/mol_C)	MeOH Selectivity (%mol_C/mol_C)	MeOH STY (mol kg_{CZZ}^{-1} h^{-1})	d_{CuO}[a] (nm)	S_{BET} (m^2/g)	H_2 Consumption[b] (mol H_2/kg_{cat})[b]
40	350	11.9	33.6	7.9	7.7	127.0	9.8
40	500	12.0	37.2	8.9	10.2	71.8	10.5
80	500	9.2	35.5	6.4	16.2	80.2	8.8
40	600	10.5	39.8	8.3	14.5	31.8	8.8

[a]Calculated using the Scherrer equation for the (111) plane of CuO.
[b]Calculated from H_2 TPR.

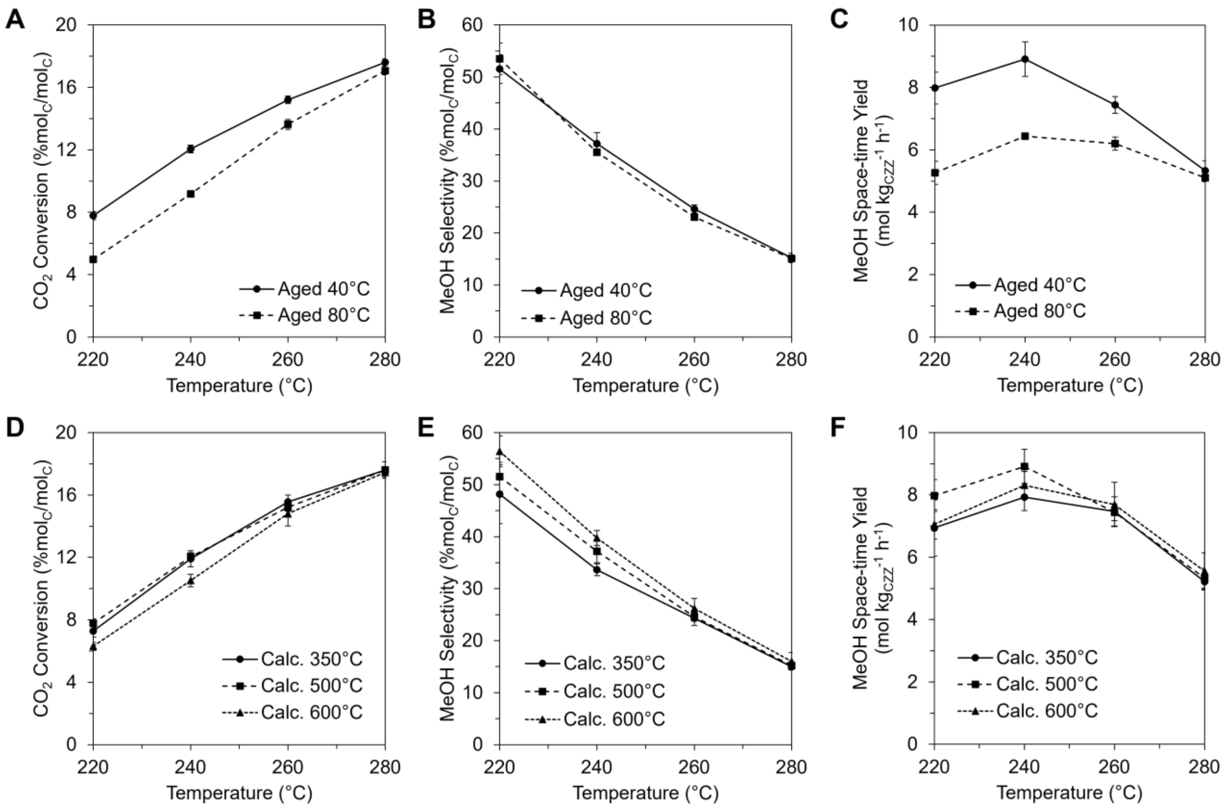

Figure S2. Catalytic performance of CZZ-613 at varied A,B,C) aging temperature (40, 80°C) and C,D,E) calcination temperature (350, 500, 600°C). Figures A and D show the CO_2 conversion while Figures B and E show the MeOH selectivity. MeOH STY are shown in Figures C and F. Base reaction conditions: 260°C, 500 psig, 18000 mL g_{CZZ}^{-1} h^{-1}, H_2:CO_2 ratio = 3:1, mass of CZZ = 0.5 g.

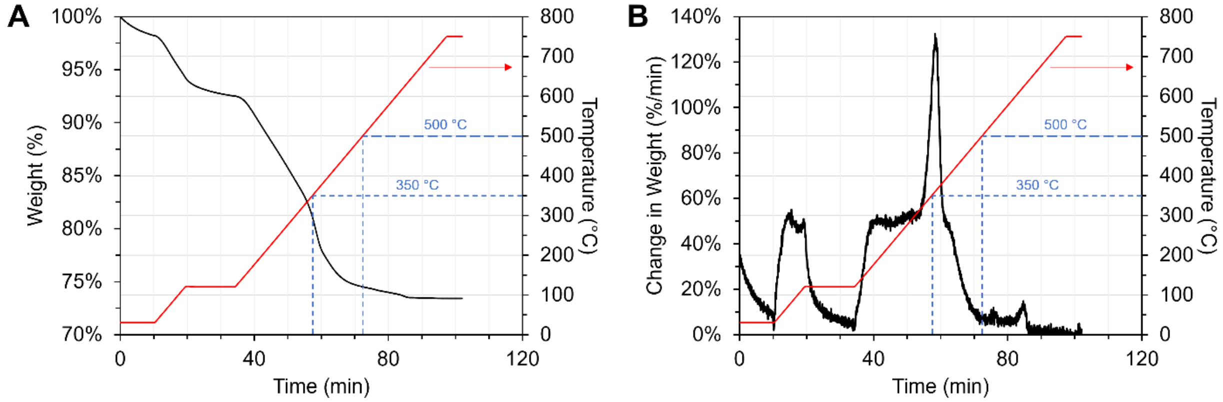

Figure S3. TGA of dried CZZ-613 plotted by A) weight and B) change in weight.

S3. <u>Effect of Composition on CZZ Activity</u>

S3.1. Theoretical CZZ compositions

Table S5. Theoretical compositions of CZZ, CuO/SiO_2, and $ZnZrO_x$ used in the study.

Notation	Cu (%mol/mol)*	Zn (%mol/mol)*	Zr (%mol/mol)*
CZZ-253	20	50	30
CZZ-433	40	30	30
CZZ-611	75	12.5	12.5
CZZ-613	60	10	30
CZZ-615	50	8.3	41.7
CuO/SiO_2	48.6	-	-
$ZnZrO_x$	-	50	50

*Relative to total moles of Cu, Zn, Zr, and Si.

S3.2. Reactivity

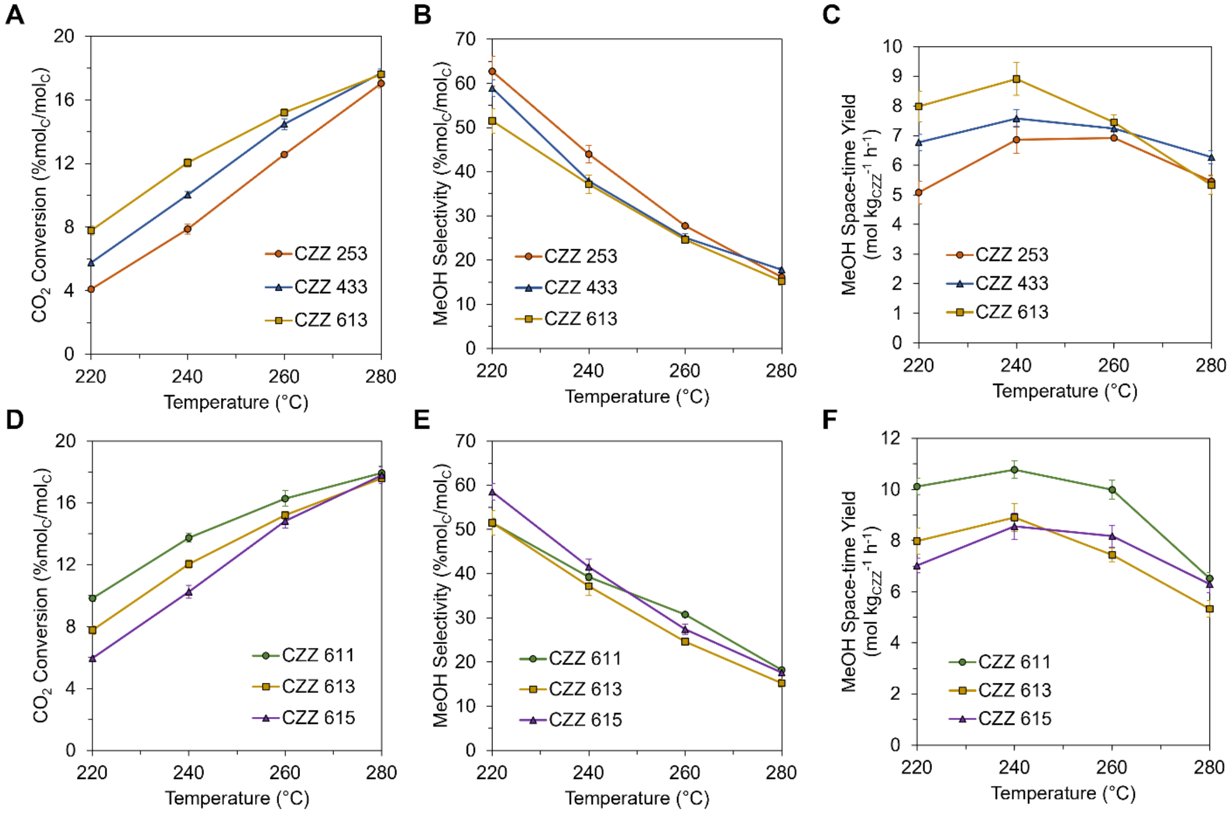

Figure S4. Catalytic performance of CZZ at varied A,B,C) Cu:Zn molar ratio and C,D,E) Zr content. Figures A and D show the CO_2 conversion while Figures B and E show the MeOH

selectivity. MeOH STY are shown in Figures C and F. Base reaction conditions: 260°C, 500 psig, 18000 mL g_{CZZ}^{-1} h^{-1}, H_2:CO_2 ratio = 3:1, mass of CZZ = 0.5 g.

S3.3. H_2-TPR

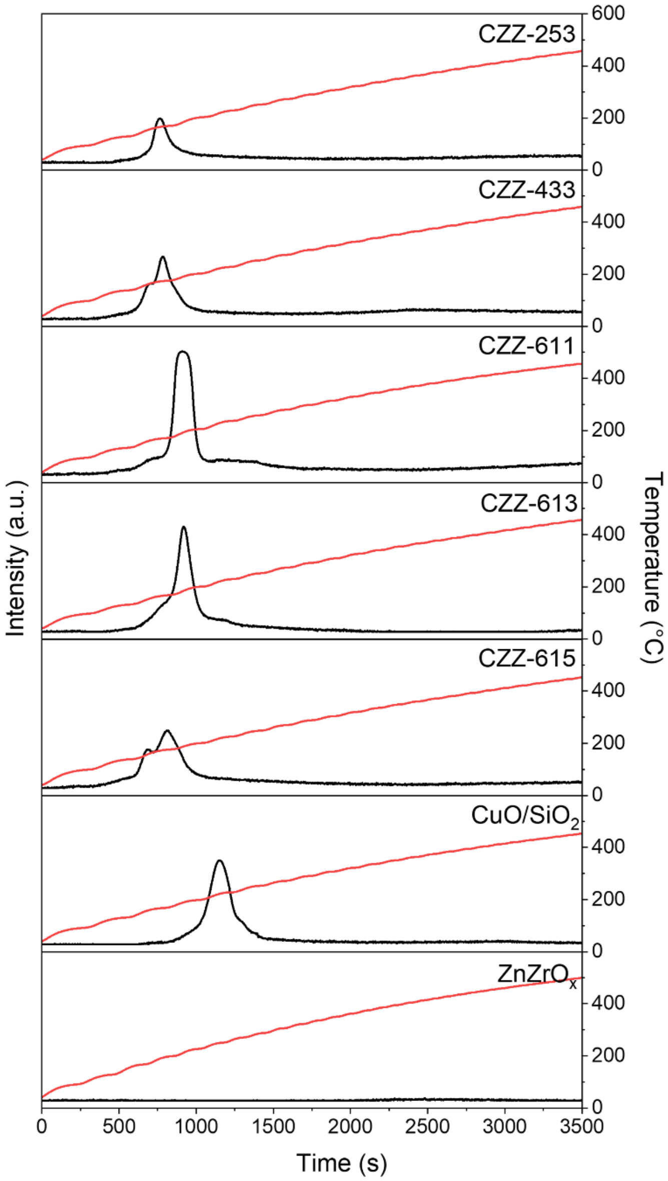

Figure S5. H_2-TPR Profiles of CZZ with different molar compositions. The catalysts are denoted as CZZ-XYZ where X:Y:Z refers to the Cu:Zn:Zr molar ratio.

S3.4. CO_2-TPD

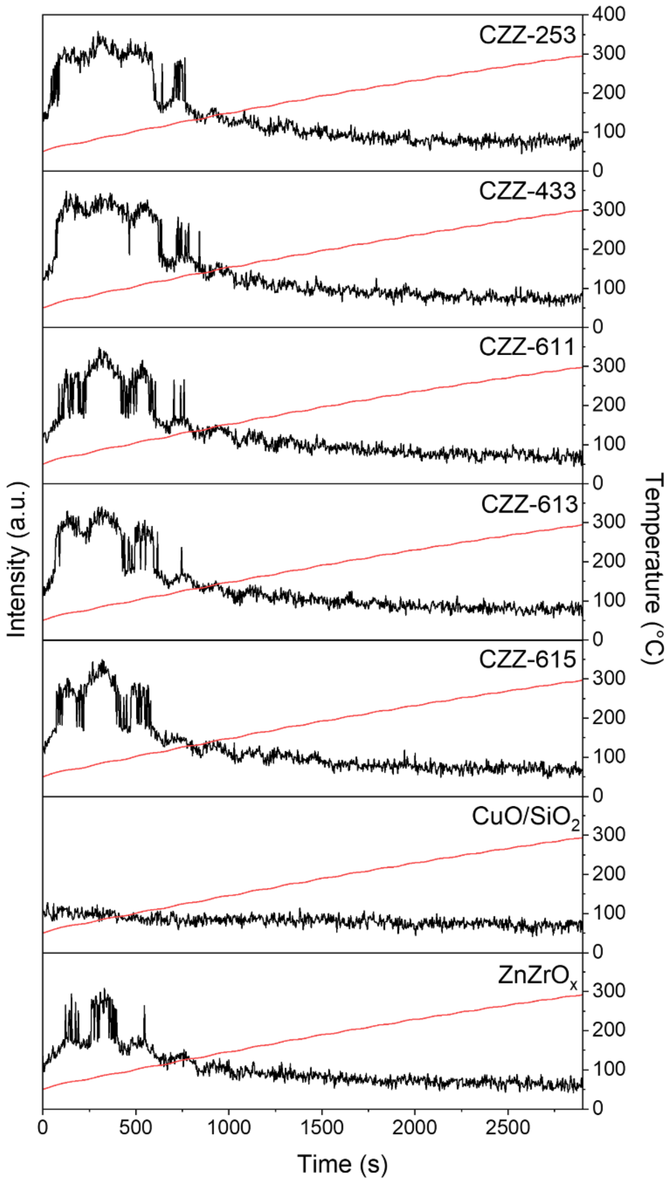

Figure S6. CO_2-TPD profiles of the calcined catalysts. The catalysts are denoted as CZZ-XYZ where X:Y:Z refers to the Cu:Zn:Zr molar ratio.

S4. <u>Tandem Catalyst Studies</u>

S4.1. External mass transfer limitation

External mass transfer limitation was evaluated by increasing the linear velocity of gases through the catalyst bed while maintaining a constant GHSV. Increasing the linear velocity improves external mass transfer of species and should thus improve activity for external mass transfer limited systems. The results shown in **Figure S7** confirm minimal change in activity even with varying linear velocities which suggest that the CZZ/SAPO-34 system is not significantly externally mass transfer limited.

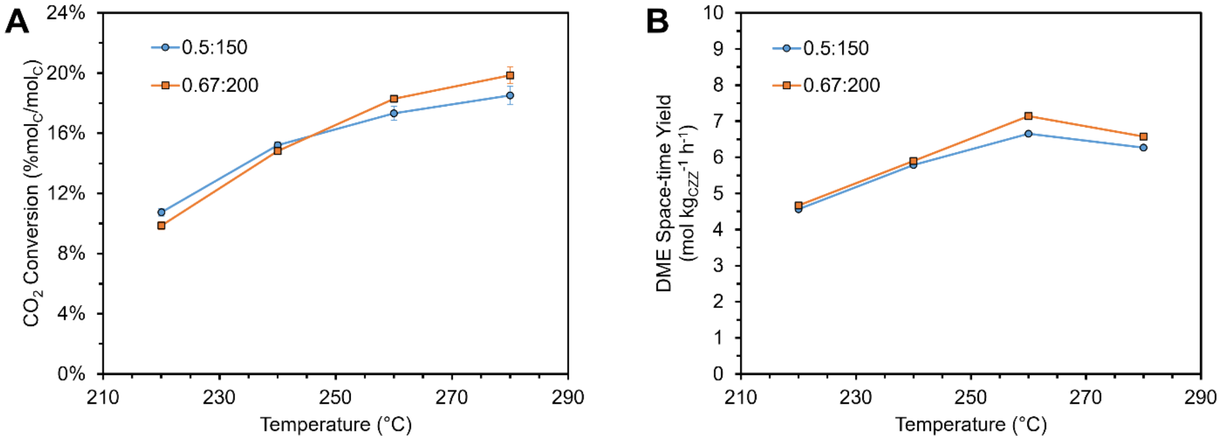

Figure S7. A) CO_2 conversion and B) DME space-time yield of CZZ-611/SAPO-34 at different linear velocities. Reaction conditions: 260°C, 500 psig, 18000 mL g_{CZZ}^{-1} h^{-1}, H_2:CO_2 ratio = 3:1, CZZ:SAPO-34 mass ratio = 1:1.

S4.2. Internal mass transfer limitation

Internal mass transfer limitation was evaluated by using smaller granule sizes for CZZ and SAPO-34. The catalysts were sieved into 60-80 mesh and compared with the performance of catalysts sieved at 30-60 mesh. An increased activity with smaller particle sizes would indicate

an internally mass transfer limited reaction. **Figure S8** shows the activity of the catalyst at different granule sizes wherein no significant differences were observed. It is therefore safe to assume that the CZZ-SAPO-34 system is not internally mass transfer limited.

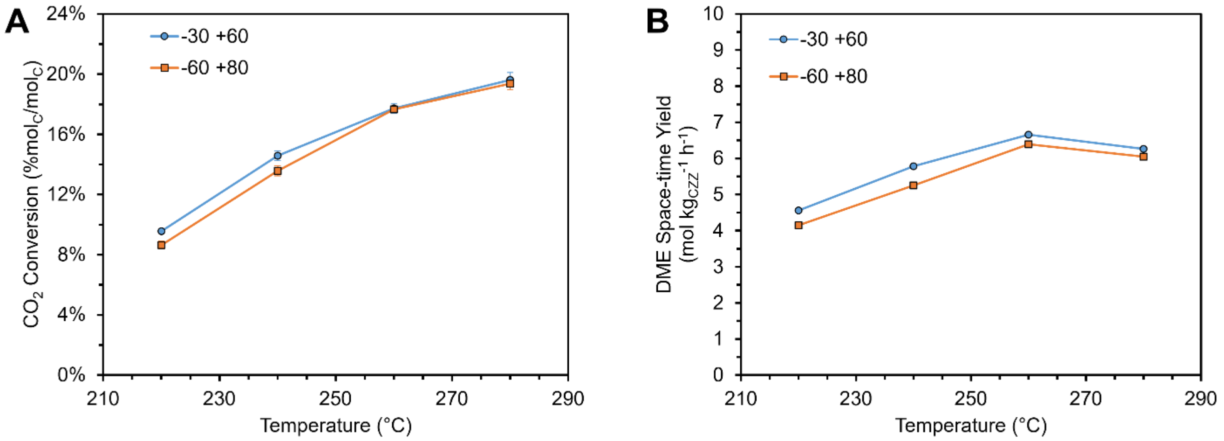

Figure S8. A) CO_2 conversion and B) DME space-time yield of CZZ-611/SAPO-34 at different particle sizes. Reaction conditions: 260°C, 500 psig, 18000 mL g_{CZZ}^{-1} h^{-1}, H_2:CO_2 ratio = 3:1, mass of CZZ = 0.5 g, CZZ:SAPO-34 mass ratio = 1:1.

S4.3. TGA of SAPO-34 used in stability test

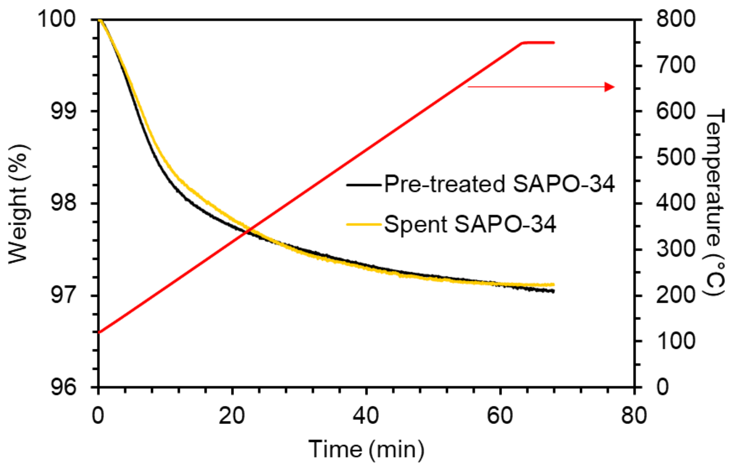

Figure S9. Thermogravimetric analysis (TGA) of the pre-treated and spent SAPO-34 catalyst.

S5. <u>Kinetic model fitting</u>

Table S6. Set of experimental tests used for the kinetic model.

Experiment No.	Temperature (°C)	Pressure (psig)	GHSV (mL g_{CZZ}^{-1} h^{-1})	H_2:CO_2 Feed Ratio	CZZ:SAPO-34 Mass Ratio
1	220	300	18000	3:1	2:1
2	240	300	18000	3:1	2:1
3	260	300	18000	3:1	2:1
4	280	300	18000	3:1	2:1
5	220	300	18000	3:1	1:1
6	240	300	18000	3:1	1:1
7	260	300	18000	3:1	1:1
8	280	300	18000	3:1	1:1
9	220	300	18000	3:1	1:2
10	240	300	18000	3:1	1:2
11	260	300	18000	3:1	1:2
12	280	300	18000	3:1	1:2
13	260	100	18000	3:1	1:1
14	260	300	18000	3:1	1:1
15	260	500	2000	3:1	1:1
16	260	500	6000	3:1	1:1
17	260	500	12000	3:1	1:1
18	260	500	24000	3:1	1:1
19	260	500	18000	1:1	1:1
20	260	500	18000	2:1	1:1
21	260	500	18000	4:1	1:1
22	260	500	18000	5:1	1:1
23	220	500	2000	3:1	2:1
24	240	500	2000	3:1	2:1
25	260	500	2000	3:1	2:1
26	280	500	2000	3:1	2:1

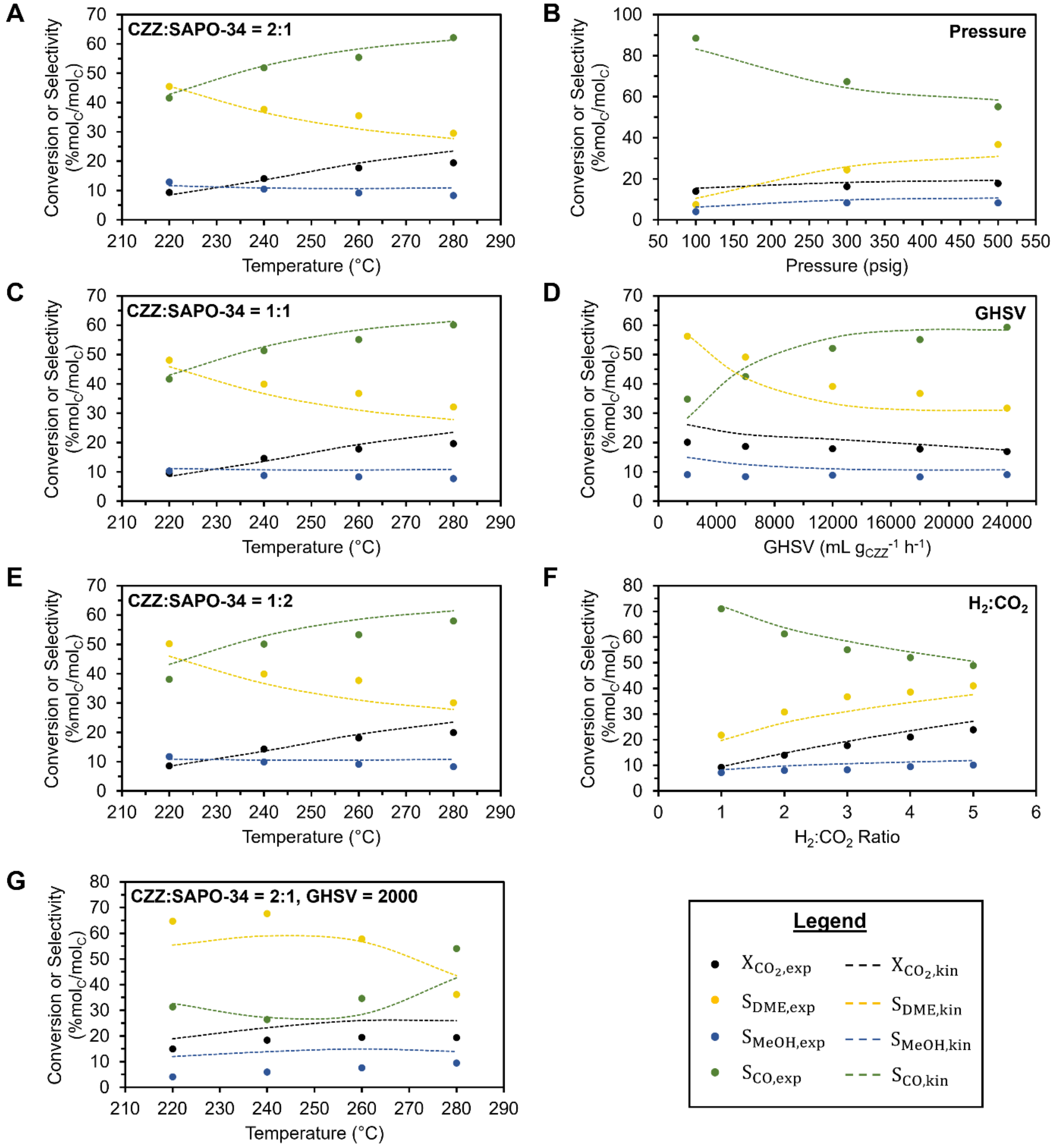

Figure S10. Comparison of experimental data vs simulated data from kinetic model. A, C, E) Activity at 220°C to 280°C with CZZ:SAPO-34 ratio of 2:1, 1:1 , and 1:2, respectively (Granule-mixed, 500 psig, 18000 mL g_{CZZ}^{-1} h^{-1}, H_2:CO_2 = 3:1, mass of CZZ = 0.5 g). B) Activity at varying pressures from 100 psig to 500 psig (Granule-mixed, 260°C, 18000 mL g_{CZZ}^{-1} h^{-1},

H_2:CO_2 = 3:1, CZZ:SAPO-34 = 1:1, mass of CZZ = 0.5 g). D) Activity at varying GHSV from 2000 mL g_{CZZ}^{-1} h^{-1} to 24000 mL g_{CZZ}^{-1} h^{-1} (Granule-mixed, 260°C, 500 psig, H_2:CO_2 = 3:1, CZZ:SAPO-34 = 1:1, mass of CZZ = 0.5 g). F) Activity at varying H_2:CO_2 ratio from 1:1 to 5:1 (Granule-mixed, 260°C, 500 psig, 18000 mL g_{CZZ}^{-1} h^{-1}, CZZ:SAPO-34 = 1:1, mass of CZZ = 0.5 g). G) Activity at 220°C to 280°C with 2000 mL g_{CZZ}^{-1} h^{-1} and CZZ:SAPO-34 = 2:1 (Granule-mixed, 500 psig, H_2:CO_2 = 3:1, mass of CZZ = 0.5 g). Circles represent experimental data while dotted lines show simulated data from kinetic model. Yellow, blue, and green colors represent DME, MeOH, and CO selectivity, respectively, while black shows CO_2 conversion.

S6. <u>Process model</u>

S6.1. Detailed process description

CO_2 (S1) and H_2 (S2) enter the plant at 30 bar and 25°C and are compressed to 35 bar through compressors C1 and C2, respectively. These are mixed with the recycled unreacted gases (S30) and heated to 260°C by flowing with the effluent from the reactor (S8) through HX1 and HP-steam through HX2. CO_2 is converted to DME in a packed bed reactor (PBR1) operating at 260°C and 35 bar. The product gases (S8) pass through a series of heat exchangers where heat generated or lost in other parts of the process was used to reduce heating/cooling requirements from utilities. S8 initially loses heat in HX1 and passes through HX3 where it is cooled to 35°C with cooling water. Cold streams (S14 and S15) from the flash drum (F1) are used in HX4 and HX5 to further reduce the temperature of the product gases. At the end of the heat exchanger series (HX6), a refrigerant is used to lower the temperature further to -55°C. The bulk of the gases (>99% H_2 and ~57% CO_2) are separated in F1 as a vapor phase stream (S14) and is recycled back to the reactor, while a liquid stream containing DME, MeOH, and H_2O and the balance of CO_2 goes out through the bottom (S15). S15 is initially heated to 110°C by going

through HX4 and HX7 before entering the first distillation tower (DT1). DT1 operates at a pressure of 35 bar and temperature range of 32°C-225°C to separate CO_2 and DME at the top (S18) and MeOH and H_2O at the bottom (S19). The CO_2/DME stream is fed to the second distillation tower (DT2), operating at 35 bar and a temperature range of 0°C-102°C. CO_2 (>99.6% purity) goes out at the top (S30) of D-102 while fuel-grade DME (>99.9% purity) is recovered at the bottom (S33). MeOH and H_2O from S25 were depressurized to 10 bar and separated in the third column (DT3) operating at a temperature range of 134°C-180°C. Industrial-grade methanol (>99.5% purity) is recovered at the top of the column (S23) and water (>99.4% purity) goes out at the bottom (S24). The separated bulk gases (S25) and CO_2 (S20) are recycled back into the reactor with 1% of the combined stream purged as gas waste (S29).

S6.2. Process stream data

Table S7. Process stream properties and flow rates.

Streams	Temperature (°C)	Pressure (bar)	Molar Vapor Fraction	Mole Flow (kmol hr^{-1})	Mass Flow (kg hr^{-1})
S1	25.00	30.00	1.00	143.25	6304.35
S2	25.00	30.00	1.00	429.75	866.32
S3	38.15	35.00	1.00	143.25	6304.35
S4	40.97	35.00	1.00	429.75	866.32
S5	32.54	35.00	1.00	2917.57	36286.46
S6	237.03	35.00	1.00	2917.57	36286.46
S7	245.00	35.00	1.00	2917.57	36286.46
S8	257.03	34.08	1.00	2643.31	36286.57
S9	99.02	34.08	0.95	2643.31	36286.57
S10	35.00	34.08	0.92	2643.31	36286.57
S11	-10.50	34.08	0.92	2643.31	36286.57
S12	-33.21	34.08	0.92	2643.31	36286.57
S13	-55.00	34.08	0.92	2643.31	36286.57
S14	-55.00	34.09	1.00	2138.29	19373.75
S15	-55.00	34.09	0.00	504.87	16910.69
S16	20.00	34.09	0.29	504.87	16910.69
S17	110.00	34.09	0.61	504.87	16910.69
S18	35.13	34.01	1.00	284.25	12536.59
S19	223.49	34.01	0.00	220.62	4374.10
S20	0.44	33.93	1.00	229.97	10036.15

S21	101.02	34.12	0.00	54.28	2500.44
S22	166.86	10.00	0.17	220.62	4374.10
S23	134.58	9.86	1.00	27.85	892.84
S24	179.85	10.04	0.00	192.77	3481.26
S25	-20.50	34.09	1.00	2138.29	19373.75
S26	-21.61	34.09	1.00	2368.26	29409.90
S27	30.00	34.09	1.00	2368.26	29409.90
S28	30.00	34.09	1.00	2344.58	29115.80
S29	30.00	34.09	1.00	23.68	294.10
S30	32.55	35.00	1.00	2344.58	29115.80

Table S8. Process stream molar compositions (%mol/mol).

Streams	CO_2	H_2	CO	H_2O	MeOH	DME
S1	0.000	0.000	0.000	0.000	0.000	0.000
S2	1.000	0.000	0.000	0.000	0.000	0.000
S3	0.000	1.000	0.000	0.000	0.000	0.000
S4	1.000	0.000	0.000	0.000	0.000	0.000
S5	0.000	1.000	0.000	0.000	0.000	0.000
S6	0.231	0.743	0.023	0.000	0.000	0.002
S7	0.231	0.743	0.023	0.000	0.000	0.002
S8	0.231	0.743	0.023	0.000	0.000	0.002
S9	0.203	0.664	0.026	0.073	0.011	0.023
S10	0.203	0.664	0.026	0.073	0.011	0.023
S11	0.203	0.664	0.026	0.073	0.011	0.023
S12	0.203	0.664	0.026	0.073	0.011	0.023
S13	0.203	0.664	0.026	0.073	0.011	0.023
S14	0.203	0.664	0.026	0.073	0.011	0.023
S15	0.146	0.820	0.032	0.000	0.000	0.002
S16	0.447	0.004	0.001	0.381	0.056	0.112
S17	0.447	0.004	0.001	0.381	0.056	0.112
S18	0.447	0.004	0.001	0.381	0.056	0.112
S19	0.793	0.007	0.002	0.000	0.000	0.198
S20	0.000	0.000	0.000	0.871	0.129	0.000
S21	0.981	0.008	0.002	0.000	0.000	0.009
S22	0.000	0.000	0.000	0.000	0.000	1.000
S23	0.000	0.000	0.000	0.871	0.129	0.000
S24	0.000	0.000	0.000	0.000	0.998	0.001
S25	0.000	0.000	0.000	0.997	0.003	0.000
S26	0.146	0.820	0.032	0.000	0.000	0.002
S27	0.227	0.741	0.029	0.000	0.000	0.003
S28	0.227	0.741	0.029	0.000	0.000	0.003
S29	0.227	0.741	0.029	0.000	0.000	0.003
S30	0.227	0.741	0.029	0.000	0.000	0.003

S7. Economic analysis

S7.1. CAPEX estimations

Table S9. Breakdown of CAPEX estimates.

	Value ($M)
Direct Costs (DC)	
Purchased equipment cost	13.7
Purchased equipment installation	6.5
Instrumentation and controls	4.9
Piping	9.3
Electrical system	1.5
Building (including services)	2.5
Yard Improvements	1.4
Service facilities	9.7
Total direct costs	49.5
Indirect Costs (IC)	
Engineering and supervision	4.5
Construction expense	5.6
Legal expense	0.5
Contractor's fee	3.0
Contingency	6.0
Total indirect costs	19.8
**Fixed Capital Investment (FCI)**	69.3
**Working Capital (WC)**	12.2
**CAPEX**	81.5

S7.2. OPEX estimations

S7.2.1 Cost of CZZ

The price of CZZ-611 was computed using the step method[8] provided in the CatCost v1.1.0 spreadsheet.[9] The base year was set to 2022 using a PPI of 359.6 and CEPCI of 802.9. The costs of raw materials were estimated based on prices found from online sources which are summarized in **Table S10**, together with process steps/equipment assumed. Most of the prices found for the raw materials are for low quantity orders (0.001-10 kg), thus a price for bulk orders was estimated through linear regression using the relation in **Equation S1** where P refers to the material price in $/kg, and Q refers to the quantity in kg. A catalyst mass yield of 37 tons was considered. The masses of raw materials followed the same ratios used during synthesis in the

laboratory. A purchase cost of $22.98/kg CZZ was estimated with the breakdown detailed in **Table S11**.

$$\ln P = \gamma \ln Q + \ln b \tag{S1}$$

Table S10. Assumptions for CatCost.

**Raw Materials**	**Assumed Price ($/kg)**
$Cu(NO_3)_2.2.5H_2O$	1.82
$Zn(NO_3)_2.6H_2O$	0.97
$ZrO(NO_3)_2.xH_2O$	14.48
Na_2CO_3	0.29
**Process Steps/Equipment**	**Quantity**
Reactor, simple (mixing)	2
Crystallizer	2
Dryer, rotary (40-100°C)	2
Kiln, continuous indirect (300-1290°C)	2
Scrubber, Nox	1

Table S11. CZZ catalyst breakdown.

	**Unit Cost ($/kg)**
**Synthesis Costs**	
Raw Materials	12.14
Process Steps	4.98
**Overheads and Margin**	
General and Administrative	0.86
Sales, Admin., Research, Distrib.	0.90
Selling Margin	4.09
**CZZ Purchase Cost**	22.98

S7.2.2. Cost of SAPO-34

The price of SAPO-34 was based on selling prices obtained from ACS Materials, Fischer Scientific, and Novarials. An estimated price for an order quantity of 12.3 tons was obtained using **Equation S1**.

S7.2.3. Cost of Operating Labor (OL)

The number of operators was estimated using **Equation S2**. The number of operators per shift (N_{OL}) was calculated using the rounded-up value obtained from **Equation S3** where N_S refers to the number of processes which handles solids while N_{NS} refers to processes that does not handle solids.[10]

$$\text{Number of operators} = N_{OL}\left(\frac{\text{Number of total shifts per year}}{\text{Number of shifts per operator}}\right) \tag{S2}$$

$$N_{OL} = \left(6.29 + 31.7 \cdot {N_S}^2 + 0.23 \cdot N_{NS}\right)^{0.5} \tag{S3}$$

Since the plant operates for 8000 h with 3 shifts per day, a total of 1000 shifts per year was considered. The number of shifts per operator per year was computed by converting 8000 h to weeks then considering each operator to do 5 shifts per week which results in a total of 238 shifts per operator per year. An N_{OL} of 4 was computed resulting to a total of 17 operators needed for the plant. We assumed an hourly wage of \$48.56/h[11] resulting in an annual labor cost of \$1.57M computed from **Equation S4**.

$$\text{Cost}_{OL} = (\text{Hourly wage})(\text{ Number of operators})\left(\frac{8\text{ h}}{\text{shift}}\right)(\text{Number of shifts per operator}) \tag{S4}$$

S8. <u>Comparison of Single Reactor and Two-Reactor System</u>

A simplified CO_2 to DME process model utilizing shortcut distillation columns (DSTWU) was modelled in Aspen Plus. Generation of utilities and heat integration was also omitted in the simplified model and default utilities stream were used. Two types of models were prepared – one utilizes a single reactor containing both CZZ and SAPO-34 (direct) while the other uses two reactors with CZZ in the first and SAPO-34 placed in the second (indirect). For this analysis, we focused our attention on the reactor costs and emissions (**Figure S11**). The performance

indicators, PEC, and heat duty are summarized in **Table S12**. We would like to note that the simplified model single-reactor model has slight differences compared to values from the main model presented in the manuscript due to the simplifications made, albeit these are minor.

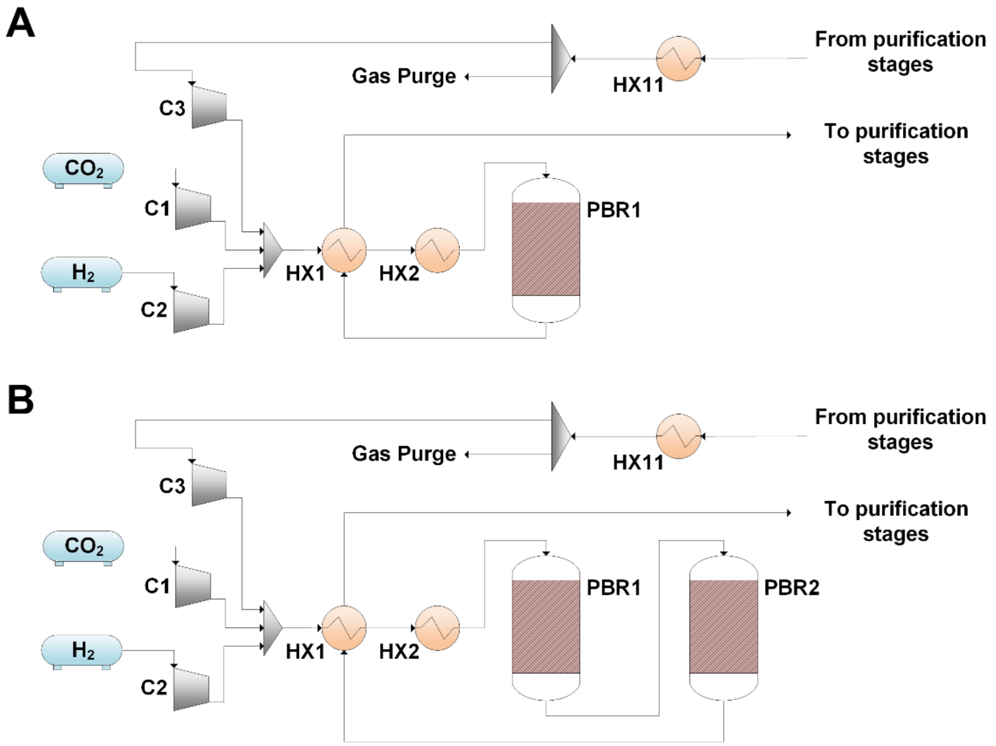

Figure S11. A) Single-reactor and B) two-reactor models considered in the analysis. The purification stages were omitted.

Table S12. Performance indicators, purchased equipment costs (PEC), and heat duty of the two-reactor and single-reactor models.

	**Units**	**Two Reactor**	**Single Reactor**
**Per pass CO_2 conversion**	$\%mol_C/mol_C$	14.6	20.6
**Per pass DME selectivity**	$\%mol_C/mol_C$	30.5	55.8
**H_2 recycle-to-feed ratio (kton/kton)**	kton/kton	6.6	4.0
**CO_2 recycle-to-feed ratio (kton/kton)**	kton/kton	5.5	3.7
**Purchased equipment costs (PEC)**			
Compressors (C1, C2, C3)	US $	3,070,700	2,977,700
Heat Exchangers (HX1, HX2, HX11)	US $	155,900	106,100
Packed Bed Reactors (PBR1, PBR2)	US $	6,097,300	3,292,400
**Heat duty**			

Heat Exchangers (HX2, HX11)*	kW	2,696	1,817
Packed Bed Reactors (PBR1, PBR2)	kW	-2,300	-2,287

*Only heat exchangers utilizing fresh steam are considered in the comparison.

References

(1) Peters, M. S.; Timmerhaus, K. D.; West, R. E. *Plant Design and Economics for Chemical Engineers*; McGraw-Hill Professional, 2002.

(2) IEA. *Global Hydrogen Review 2023*; IEA, Paris, 2023. https://www.iea.org/reports/global-hydrogen-review-2023 (accessed 2024-11-06).

(3) EIA. *How much carbon dioxide is produced per kilowatthour of U.S. electricity generation?* 2023. https://www.eia.gov/tools/faqs/faq.php?id=74&t=11 (accessed 2024 November 6).

(4) EIA. *Carbon Dioxide Emissions Coefficients*. 2024. https://www.eia.gov/environment/emissions/co2_vol_mass.php (accessed 2024 November 6).

(5) Li, L.; Mao, D.; Yu, J.; Guo, X. Highly selective hydrogenation of CO2 to methanol over CuO-ZnO-ZrO2 catalysts prepared by a surfactant-assisted co-precipitation method. *Journal of Power Sources* **2015**, *279*, 394-404. DOI: 10.1016/j.jpowsour.2014.12.142.

(6) Witoon, T.; Kachaban, N.; Donphai, W.; Kidkhunthod, P.; Faungnawakij, K.; Chareonpanich, M.; Limtrakul, J. Tuning of catalytic CO2 hydrogenation by changing composition of CuO-ZnO-ZrO2 catalysts. *Energy Conversion and Management* **2016**, *118*, 21-31. DOI: 10.1016/j.enconman.2016.03.075.

(7) Natesakhawat, S.; Lekse, J. W.; Baltrus, J. P.; Ohodnicki, P. R., Jr.; Howard, B. H.; Deng, X.; Matranga, C. Active Sites and Structure–Activity Relationships of Copper-Based Catalysts for Carbon Dioxide Hydrogenation to Methanol. *ACS Catalysis* **2012**, *2* (8), 1667-1676. DOI: 10.1021/cs300008g.

(8) Baddour, F. G.; Snowden-Swan, L.; Super, J. D.; Van Allsburg, K. M. Estimating Precommercial Heterogeneous Catalyst Price: A Simple Step-Based Method. *Organic Process Research & Development* **2018**, *22* (12), 1599-1605. DOI: 10.1021/acs.oprd.8b00245.

(9) ChemCatBio. *CatCost*. 2021. https://catcost.chemcatbio.org/home (accessed 2024 November 6).

(10) Verret, J.; Qiao, R.; Barghout, R. A. *Foundations of Chemical and Biological Engineering I*; 2020.

(11) U.S. Bureau of Labor Statistics, *Occupational Employment and Wage Statistics*. 2024. https://www.bls.gov/oes/current/oes518091.htm (accessed 2024 November 6).
